# Disparities in perioperative mortality outcomes between First Nations and non-First Nations peoples in Australia: protocol for a systematic review and planned meta-analysis

**DOI:** 10.1186/s13643-024-02611-3

**Published:** 2024-08-05

**Authors:** Edith B. Waugh, Matthew J. L. Hare, David A. Story, Lorena Romero, Mark Mayo, Heidi Smith-Vaughan, Jennifer R. Reilly

**Affiliations:** 1 Department of Anaesthesia and Perioperative Medicine, Royal Darwin Hospital, Darwin, NT Australia; 2 Menzies School of Health Research, Charles Darwin University, Darwin, NT Australia; 3 Flinders University, Adelaide, SA Australia; 4 Endocrinology Department, Royal Darwin Hospital, Darwin, NT Australia; 5 Department of Critical Care, the University of Melbourne, Melbourne, VIC Australia; 6 Department of Anaesthesia, Austin Health, Melbourne, VIC Australia; 7 The Ian Potter Library, Alfred Health, Melbourne, VIC Australia

**Keywords:** Postoperative/perioperative mortality,, Surgical procedures, Operative,, Anaesthesia,, Australia,, Outcome assessment, Health care,, Systematic reviews,, Meta-analysis,, First Nations people,, Australian Aboriginal and Torres Strait Islander peoples,, Indigenous health,, Health equity,, Health status disparities

## Abstract

**Background:**

Health inequities persist among First Nations people living in developed countries. Surgical care is pivotal in addressing a significant portion of the global disease burden. Evidence regarding surgical outcomes among First Nations people in Australia is limited. The perioperative mortality rate (POMR) indicates timely access to safe surgery and predicts long-term survival after major surgery. This systematic review will examine POMR among First Nations and non-First Nations peoples in Australia.

**Methods:**

A systematic search strategy using MEDLINE, Embase, Emcare, Global Health, and Scopus will identify studies that include First Nations people and non-First Nations people who underwent a surgical intervention under anaesthesia in Australia. The primary focus will be on documenting perioperative mortality outcomes. Title and abstract screening and full-text review will be conducted by independent reviewers, followed by data extraction and bias assessment using the ROBINS-E tool. Meta-analysis will be considered if there is sufficient homogeneity between studies. The quality of cumulative evidence will be evaluated following the Grading of Recommendations, Assessment, Development and Evaluation (GRADE) criteria.

**Discussion:**

This protocol describes the comprehensive methodology for the proposed systematic review. Evaluating disparities in perioperative mortality rates between First Nations and non-First Nations people remains essential in shaping the discourse surrounding health equity, particularly in addressing the surgical burden of disease.

**Systematic review registration:**

PROSPERO CRD42021258970.

**Supplementary Information:**

The online version contains supplementary material available at 10.1186/s13643-024-02611-3.

## Background

Globally, 313 million surgical interventions are performed annually, representing a vital component in addressing one-third of the global burden of disease [1]. Timely, safe surgical care saves lives and mitigates disability. Despite advancements in surgical care, First Nations people living in developed countries continue to experience disparate health outcomes [2].

A critical metric that serves both as an indicator of timely access to safe surgery and a validated predictor of longer-term survival after major surgical procedures is the perioperative mortality rate (POMR) [3, 4]. POMR includes all intraoperative and postoperative deaths within a specified timeframe after surgery. The 30-day or ‘early’ POMR is the most readily available and globally accepted gauge of surgical outcomes [3]. Mortality within 30 days after surgery ranks as the third leading cause of death worldwide [5]. Furthermore, ‘late’ POMR statistics are reported at intervals such as 90 days, 1 year, or 2 years or beyond, providing a comprehensive assessment of surgical outcomes over time.

Given the persistent health disparities faced by First Nations people and the impact of surgical interventions on their health and well-being, a focused review of POMR within this community in the Australian context is essential.

### First Nations people and perioperative mortality

First Nations communities, peoples, and nations, also referred to as Indigenous peoples by the United Nations, are those who have a historical continuity with pre-invasion and pre-colonial societies that developed on their territories. These groups consider themselves distinct from other sectors of the societies that now prevail on those territories [6]. Despite their unique social, cultural, economic, and political characteristics and responsibilities, and their rights to recognition of their identities, way of life, and traditional land, these rights have been historically denied [2]. Understanding the perioperative mortality rates among First Nations peoples requires acknowledging these contexts and addressing the disparities that exist in healthcare outcomes.

First Nations people living in developed countries like New Zealand and Canada experience higher POMR than non-First Nations people. In New Zealand, data from national registries indicate that the Māori population faces a 50% higher 30-day POMR following emergency laparotomy, with rates of 8.8% compared to 5.5% in the non-Māori population [7]. Additionally, Māori patients with diabetes have a significantly higher 30-day POMR after major and minor lower-limb amputation. Specifically, the hazard ratios are 1.46, 95% *CI*: 1.08–1.98 for major amputations, and 1.73 (95% *CI*: 1.02–2.94) for minor amputations compared to non-Māori patients [8]. Hospitalisation data further reveal that Māori patients were 30% more likely to die within 30 days of an elective/waiting list procedure under general anaesthesia, with a hazard ratio of 1.32 (95% *CI*: 1.20–1.50) [9]. In Canada, a systematic review synthesising data from seven studies across four cohorts showed that First Nations people experience a 30% increase in postoperative mortality compared to non-First Nations populations (pooled *HR* 1.30, 95% *CI*: 1.09–1.54) [10].

An earlier global systematic review examining the POMR among First Nations populations including Australia, New Zealand, and the United States suggested a disparity in surgical outcomes. However, that comprehensive analysis identified limitations in the quality of studies, with the main limitation being suboptimal First Nations status ascertainment [11]. Another systematic review, encompassing postoperative outcomes in paediatric surgery across the Americas and Oceania, reported finding more than a twofold higher overall 30-day POMR among First Nations children (*OR* 2.23, 95% *CI* 1.23–4.05) [12]. While informative, these two global systematic reviews had limited representation from Australia, incorporating only seven adult studies (all cardiac) and two paediatric studies (one cardiac and one liver transplant) from Australia. The diversity of First Nations populations in Australia cannot be underestimated, spanning over 250 different language groups. First Nations peoples have lived on this continent for over 60,000 years and now constitute 3.8% of the population [13].

Consequently, the generalisability of the findings from these global reviews to other surgical cohorts across diverse First Nations populations in Australia is unclear. Thus, a focused and comprehensive review specifically addressing POMR among First Nations people in Australia is required. In this manuscript, we respectfully use the term ‘First Nations people’ for the diverse Aboriginal and/or Torres Strait Islander populations across Australia.

Factors associated with increased perioperative mortality include advanced age, high comorbid disease burden, and emergency surgery [14]. However, the 2016 Royal Australasian College of Surgeons national surgical mortality audit highlighted the 23-year difference in the median age of death for First Nations people compared with non-First Nations people (55 vs. 78 years old at the time of death) [15]. In Australia, First Nations people experience a disease burden 2.2 times the rate of non-First Nations people [16], contributing to First Nations people being hospitalised at 2.6 times increased rate [17], but less likely to receive a medical or surgical procedure in hospital [18]. The rate of elective surgery for First Nations Australians was lower than for non-First Nations Australians (61 and 82 per 1000 population) [18]. First Nations Australians also waited longer for admission from elective surgery waiting lists than non-First Nations Australians, with median waiting times of 50 and 39 days respectively [19]. The rate of emergency admissions involving surgery for First Nations Australians was twice the rate of non-First Nations Australians (27 and 13 per 1000 population, respectively) [17]. Given the systemic health inequities and distinct disease burden among First Nations people, targeted evaluation of their postsurgical outcomes in Australia is warranted.

This protocol outlines the methods for a proposed systematic review that will compare perioperative mortality outcomes between First Nations and non-First Nations people in Australia. The review will evaluate POMR, categorised into early (30 days) and late (90 days, 1 year, or 2 years or beyond).

## Methods

This systematic review protocol follows Cochrane Collaboration and Meta-analysis of Observational Studies in Epidemiology (MOOSE) guidelines [20, 21]. Additionally, the reporting adheres to the Preferred Reporting Items for Systematic Review and Meta-Analysis protocols (PRISMA-P) 2015 statement [22], to ensure comprehensive and transparent reporting (checklist attached in Appendix 1).

### Eligibility criteria

#### Study design/characteristics

All study types will be included, including longitudinal and case-cohort, prospective, and retrospective, randomised-controlled trials and observational, single, and multicentre, full-reports and conference abstracts, reporting on mortality outcomes associated with surgery.

#### Timeframe

Studies that report early perioperative mortality outcomes (during surgery, admission, and up to 30-day post-surgery) and late postoperative mortality outcomes (later than 30-day post-surgery) at any other specified time will be included.

#### Participants

Studies will be considered for inclusion if they include First Nations patients of any age undergoing emergency or elective surgical procedures in an operating theatre environment and are performed under anaesthesia at a healthcare facility in Australia. Day cases and procedures requiring inpatient admission will be included. Minimally invasive procedures not requiring an operating theatre and not performed under anaesthesia, such as percutaneous radiological and percutaneous cardiac procedures, will not be included.

#### Exposure/comparators and eligibility criteria

In assessing disparities between First Nations (including Aboriginal and/or Torres Strait Islander, Indigenous, and other terms) and non-First Nations populations in the context of surgical procedures in Australia, we rely on identifying First Nations status to categorise individuals into these groups. This categorisation forms the basis of our comparative analysis. To be eligible for inclusion in our review, studies must compare outcomes between individuals identifying as First Nations people of Australia and outcomes in non-First Nations people in Australia.

#### Outcomes

Studies will be included if mortality outcomes (numbers or rates of death) are reported on a timeline respective to surgery: during surgery, admission, up to 30-day post-surgery, or any time post-surgery where the elapsed time from surgery was documented.

#### Setting and language

The included studies must represent surgeries performed in Australia. Only articles reported in English will be included.

### Information sources

Five major electronic bibliographic databases, MEDLINE, Embase, Emcare, Global Health, and Scopus, will be searched for relevant articles using predefined systematic strategies (see the ‘Search strategy’). To maximise search outcomes, the electronic databases will be supplemented by a manual search of the reference lists of all included studies to check for additional potentially relevant studies. Forward citation tracking will also be added to check for other possible studies. Authors will be contacted for supplemental information when published data are incomplete.

Grey literature searching will be conducted using a combination of resources such as Google Scholar, government and agency websites (Australian Bureau of Statistics (ABS), Australian Institute of Health and Welfare (AIHW)), non-governmental organisation websites (Australian Indigenous HealthInfoNet, professional medical associations, university repositories), and consultation with experts in First Nations peoples’ health and surgical outcomes.

The International Prospective Register of Systematic Reviews (PROSPERO) will be periodically checked for any relevant systematic review projects for perioperative mortality in First Nations people in Australia [23].

### Search strategy

Following the development and piloting of the search strategy, the final searches will be conducted by an experienced research librarian on five databases: Ovid MEDLINE (Medical Literature Analysis and Retrieval System Online), Ovid Embase (Excerpta Medica Database), Ovid Emcare, Ovid Global Health, and Scopus. The search strategy will use a combination of database-specific subject headings and free text terms that will cover three concept areas: Concept A, operative surgical procedures including generic and specific modes; Concept B, mortality/death/survival/life expectancy outcomes; and Concept C, broad and specific First Nations language groups in Australia.

The search terms will be combined using the AND and OR Boolean operators. Before finalising the review, the search strategy will be reapplied to ensure an updated search retrieval (see Appendix 2).

### Study records

#### Data management

Literature search results will be imported to Covidence, a web-based collaboration platform for conducting systematic reviews [24]. The duplicate records will be removed.

#### Selection process

The selection of studies to be included in the review will be conducted in two stages. At first, the studies will be independently reviewed through title and abstract screening by two reviewers based on the predefined eligibility criteria. The reviewers will discuss discrepancies and involve a third-party arbitration if required.

For all studies that pass the first stage, the full articles will be uploaded to the Covidence database. Two reviewers will again conduct the full-text review. Any discrepancies will be resolved through discussion and involvement of a third-party arbitration as required. The reasons for excluding each study will be recorded during the full-text screening process.

#### Data collection process

All selected studies will have data extracted by the primary reviewer as per the pre-specified data extraction template. The template will be piloted on the first three selected studies, to ensure reliability and validity, and adjusted as necessary. Biostatistician expertise will be utilised to clarify data fields essential for statistical analyses.

Data extraction will be performed in duplicate by two reviewers independently. Any variations will be discussed, and a third party will adjudicate unresolved disagreements. Authors of studies may be contacted if required to improve completeness.

If multiple reports of a single study (same population, same study period, and overlapping procedures, e.g. all cardiac vs. cardiac valve replacement) are identified, the study with the most inclusive population (i.e. all cardiac) will be included in the final review.

### Data items

The Population, Exposure/Intervention, Comparator, Outcomes, and Study characteristics (PECOS) framework will be used for each study to systematise the extraction, as per the data collection process, to ensure consistency.

### Outcomes and prioritisation

The outcome is the difference in POMR post-surgery in First Nations and non-First Nations people. This includes crude POMR, defined as the unadjusted mortality rates and adjusted POMR, which accounts for potential confounders such as age, sex, and comorbidities. Mortality will be measured across various timeframes: intraoperative (during surgery), during admission (before discharge), 30-day postoperative, and long-term postoperative (any time beyond 30-day post-surgery, if documented). Studies reporting crude or adjusted perioperative mortality rates and their difference with 95% confidence intervals will be summarised. A meta-analysis will be considered if the included studies demonstrate sufficient accuracy and homogeneity in quality and reporting standards.

Mortality is prioritised as it provides a clear, measurable endpoint essential for evaluating disparities in surgical outcomes between different population groups.

### Risk of *bias* in individual studies

Two reviewers will independently conduct the risk-of-bias assessment and arbitrate any unresolved differences by a third researcher. The tool for evaluating the risk of bias, when reviewing the methodological quality of the studies, will be the ROBINS-E assessment tool (Risk of Bias In Non-randomised Studies — in Exposures) [25]. Although RCTs are included in this review, they will be treated as observational cohorts to extract data relevant to the disparities in postoperative mortality outcomes between First Nations and non-First Nations participants. This allows the use of ROBINS-E across all study types, focusing on the observational data rather than the intervention effects. The tool covers seven domains, including bias due to confounding, bias arising from the measurement of exposure (comparator factor, i.e. ascertainment of First Nations status), bias in the selection of participants into the study (surgical candidate), bias due to postexposure intervention (follow-up regimen effect), bias due to missing data, bias arising from the measurement of outcome (attrition bias), and bias in the selection of the reported results. Each domain will be assigned a risk-of-bias category from the following: ‘low risk’ of bias, ‘some concerns of bias’, ‘high risk’ of bias, and ‘very high risk’ of bias. The scores of each domain lead to overall risk-of-bias judgement.

### Data synthesis

We will categorise the risk data into surgical specialties. We will categorise the outcomes data into ‘early perioperative mortality’ (death within 30 days of surgery, including during admission) and ‘late postoperative mortality’ (death after 30-day post-surgery).

A narrative synthesis will be provided in tables and text to summarise study characteristics and outcomes. In the instance of missing data, the authors of the relevant study will be contacted. Meta-analysis may be performed for early and/or late mortality studies if studies are sufficiently homogeneous in design, and the pooled random effects model reports favourable statistical heterogeneity (*I*^2^ > 50%). The analysis will be completed with R statistics [26], and the results will be displayed as a forest plot.

### *Meta*-*bias*(es)

The possibility of publication bias and outcome reporting bias will be explored by examining the characteristics of included studies, including published conference study abstracts, study protocols, data supplied by authors on request for additional information, and published peer-reviewed manuscripts.

Additionally, funnel plots [27] will be created for each meta-analysis to visually inspect for asymmetry, with asymmetrical plots suggesting the presence of publication bias. We will conduct Egger’s test [28] to detect asymmetry in the funnel plot, which can indicate the presence of publication bias. As an alternative to Egger’s test, we will use Harbord’s test [29] for small study effects.

### Confidence in cumulative evidence

Two reviewers will assess the quality and strength of evidence for reported outcomes using the Grading of Recommendations, Assessment, Development and Evaluation (GRADE) field [30] approach. A third reviewer will evaluate possible disagreement.

### Ethics

Human research ethics committee approval is not required for this review as no original primary data will be collected.

This protocol and review are part of a body of work that plans to address health equity issues impacting First Nations people in Australia. The review will follow recommendations by the Australian Commission on Safety and Quality in Health Care [31] and the National Health and Medical Research Council (NHMRC)’s strategic framework for improving Aboriginal and Torres Strait Islander health through research [32] while ensuring adherence to NHMRC’s ethical guidelines for research with Aboriginal and Torres Strait Islander peoples [33, 34]. The authors recognise, and will seek, principles of strength-based research [35], where we focus on the strengths and resilience of First Nations people to examine and address challenges and avoid deficit-based perspective where First Nations status is pathologised. This research is conducted in consultation and collaboration with First Nations people. It will be critically appraised using the CREATE Aboriginal and Torres Strait Islander quality appraisal tool [36] to consider culturally responsive reporting.

## Discussion

This protocol describes the comprehensive approach to our proposed systematic review, with a search strategy for five major databases, including published peer-reviewed journals and conference abstracts. Grey literature, including government and national reports and data repositories, will also be explored to minimise publication and reported outcome bias.

Evaluating disparities in perioperative mortality rates between First Nations and non-First Nations peoples remains essential in shaping the discourse surrounding health equity, particularly in addressing the surgical burden of disease. The review will aim to bridge knowledge gaps and advocate for enhanced healthcare systems that address inequities for First Nations peoples in Australia.

A focused review of POMR outcomes in First Nations peoples in Australia will provide a deeper understanding of these communities’ unique healthcare needs and challenges. This understanding of the magnitude of disparity in POMR in Australia has implications for guiding health equity-related policies and the well-being of First Nations communities in Australia.

### Supplementary Information

Appendix 1. PRISMA-P Checklist.

Appendix 2. Search.

## Data Availability

N/A.

## Abbreviations

ABS: Australian Bureau of Statistics
AIHW: Australian Institute of Health and Welfare
GRADE: Grading of Recommendations, Assessment, Development and Evaluation
PECOS: Population, Exposure, Comparator, Outcomes, and Study characteristics framework
POMR: Perioperative mortality rate
PRISMA-P: Preferred Reporting Items for Systematic Review and Meta-Analysis Protocol
PROSPERO: The International Prospective Register of Systematic Reviews
ROBINS-E: Risk of Bias In Non-randomised Studies — in Exposures
MOOSE: Meta-analysis of Observational Studies in Epidemiology
NHMRC: National Health and Medical Research Council
