# Supplementary material for: Disparities in perioperative mortality outcomes between First Nations and non-First Nations peoples in Australia: protocol for a systematic review and planned meta-analysis
Source: Syst Rev. 2024 Aug 5;13:208. doi: 10.1186/s13643-024-02611-3 (PMC11299354; doi:10.1186/s13643-024-02611-3)
Supplement: Supplementary file 2 — Appendix 2. Search. [file 13643_2024_2611_MOESM2_ESM.docx]

Database(s): **Ovid MEDLINE(R)** Search Strategy:

| **#** | **Searches** |
| --- | --- |
| 1 | surgical procedures, operative/ or ablation techniques/ or cautery/ or cryosurgery/ or corneal surgery, laser/ or lithotripsy, laser/ or anastomosis, surgical/ or gastroenterostomy/ or pancreaticojejunostomy/ or salpingostomy/ or vasovasostomy/ or bariatric surgery/ or lipectomy/ or lipoabdominoplasty/ or biopsy/ or curettage/ or "dilatation and curettage"/ or vacuum curettage/ or cytoreduction surgical procedures/ or debridement/ or decompression, surgical/ or microvascular decompression surgery/ or device removal/ or digestive system surgical procedures/ or anastomosis, roux-en-y/ or appendectomy/ or biliopancreatic diversion/ or cholecystectomy/ or cholecystectomy, laparoscopic/ or cholecystostomy/ or choledochostomy/ or portoenterostomy, hepatic/ or sphincterotomy, endoscopic/ or sphincterotomy, transduodenal/ or colectomy/ or proctocolectomy, restorative/ or endoscopy, digestive system/ or cholangiopancreatography, endoscopic retrograde/ or endoscopy, gastrointestinal/ or balloon enteroscopy/ or double-balloon enteroscopy/ or single-balloon enteroscopy/ or colonoscopy/ or sigmoidoscopy/ or duodenoscopy/ or endoscopic mucosal resection/ or esophagoscopy/ or gastroscopy/ or proctoscopy/ or transanal endoscopic surgery/ or transanal endoscopic microsurgery/ or pyloromyotomy/ or enterostomy/ or cecostomy/ or colostomy/ or duodenostomy/ or ileostomy/ or jejunostomy/ or esophagectomy/ or esophagoplasty/ or esophagostomy/ or fundoplication/ or gastrectomy/ or gastropexy/ or gastroplasty/ or gastrostomy/ or heller myotomy/ or hemorrhoidectomy/ or hepatectomy/ or jejunoileal bypass/ or lateral internal sphincterotomy/ or liver transplantation/ or pancreas transplantation/ or pancreatectomy/ or pancreaticoduodenectomy/ or proctectomy/ or elective surgical procedures/ or electrosurgery/ or endocrine surgical procedures/ or adrenalectomy/ or castration/ or orchiectomy/ or ovariectomy/ or salpingo-oophorectomy/ or hypophysectomy/ or parathyroidectomy/ or pinealectomy/ or thyroidectomy/ or fasciotomy/ |
| 2 | hemostasis, surgical/ or keratectomy/ or corneal surgery, laser/ or keratectomy, subepithelial, laser-assisted/ or keratomileusis, laser in situ/ or photorefractive keratectomy/ or laparotomy/ or ligation/ or lymph node excision/ or neck dissection/ or mastectomy/ or metastasectomy/ or microsurgery/ or minimally invasive surgical procedures/ or monitoring, intraoperative/ or myotomy/ or pyloromyotomy/ or sphincterotomy/ or obstetric surgical procedures/ or colposcopy/ or colpotomy/ or culdoscopy/ or cesarean section/ or hysteroscopy/ or hysterotomy/ or neurosurgical procedures/ or anterior temporal lobectomy/ or brain tissue transplantation/ or cerebrospinal fluid shunts/ or ventriculoperitoneal shunt/ or ventriculostomy/ or craniotomy/ or decompressive craniectomy/ or trephining/ or denervation/ or parasympathectomy/ or vagotomy/ or sympathectomy/ or ganglionectomy/ or cordotomy/ or nerve crush/ or rhizotomy/ or foraminotomy/ or hypophysectomy/ or laminectomy/ or laminoplasty/ or microvascular decompression surgery/ or stereotaxic techniques/ or radiosurgery/ |
| 3 | ophthalmologic surgical procedures/ or blepharoplasty/ or dacryocystorhinostomy/ or eye enucleation/ or eye evisceration/ or sclerostomy/ or trabeculectomy/ or iridectomy/ or orbit evisceration/ or refractive surgical procedures/ or cataract extraction/ or capsulorhexis/ or phacoemulsification/ or corneal transplantation/ or descemet stripping endothelial keratoplasty/ or epikeratophakia/ or keratoplasty, penetrating/ or corneal surgery, laser/ or keratectomy, subepithelial, laser-assisted/ or keratomileusis, laser in situ/ or photorefractive keratectomy/ or keratotomy, radial/ or lens implantation, intraocular/ or posterior capsulotomy/ or scleroplasty/ or scleral buckling/ or vitrectomy/ or vitreoretinal surgery/ or oral surgical procedures/ or apicoectomy/ or gingivectomy/ or gingivoplasty/ or glossectomy/ or jaw fixation techniques/ or orthognathic surgical procedures/ or osteotomy, le fort/ or osteotomy, sagittal split ramus/ or orthopedic procedures/ or acetabuloplasty/ or amputation/ or disarticulation/ or hemipelvectomy/ or arthrodesis/ or spinal fusion/ or arthroplasty/ or anterior cruciate ligament reconstruction/ or bone-patellar tendon-bone grafting/ or arthroplasty, replacement/ or hemiarthroplasty/ or total disc replacement/ or posterior cruciate ligament reconstruction/ or arthroscopy/ or joint capsule release/ or bone lengthening/ or ilizarov technique/ or osteogenesis, distraction/ or bone transplantation/ or cementoplasty/ or vertebroplasty/ or kyphoplasty/ or diskectomy/ or diskectomy, percutaneous/ or fracture fixation/ or closed fracture reduction/ or fracture fixation, internal/ or fracture fixation, intramedullary/ or open fracture reduction/ or laminectomy/ or limb salvage/ or meniscectomy/ or osteotomy/ or alveolar bone grafting/ or synovectomy/ or tendon transfer/ or tenodesis/ or tenotomy/ or traction/ or ulnar collateral ligament reconstruction/ or ostomy/ or cystostomy/ or enterostomy/ or cecostomy/ or colostomy/ or duodenostomy/ or ileostomy/ or jejunostomy/ or esophagostomy/ or gastrostomy/ or middle ear ventilation/ or nephrostomy, percutaneous/ or pharyngostomy/ or thoracostomy/ or tracheostomy/ or ureterostomy/ |
| 4 | otorhinolaryngologic surgical procedures/ or adenoidectomy/ or laryngectomy/ or laryngoplasty/ or laryngoscopy/ or nasal surgical procedures/ or rhinoplasty/ or neck dissection/ or otologic surgical procedures/ or auditory brain stem implantation/ or cochlear implantation/ or endolymphatic shunt/ or fenestration, labyrinth/ or mastoidectomy/ or middle ear ventilation/ or myringoplasty/ or ossicular replacement/ or stapes surgery/ or transtympanic micropressure treatment/ or tympanoplasty/ or pharyngectomy/ or pharyngostomy/ or tonsillectomy/ or tracheostomy/ or tracheotomy/ or pelvic exenteration/ or perioperative care/ or intraoperative care/ or postoperative care/ or perioperative period/ or intraoperative period/ or operative time/ or postoperative period/ or anesthesia recovery period/ or pneumonectomy/ or prophylactic surgical procedures/ or prosthesis implantation/ or maxillofacial prosthesis implantation/ or mandibular prosthesis implantation/ or reconstructive surgical procedures/ or abdominoplasty/ or lipoabdominoplasty/ or acetabuloplasty/ or dermatologic surgical procedures/ or blepharoplasty/ or rhytidoplasty/ or skin transplantation/ or herniorrhaphy/ or limb salvage/ or lipectomy/ or mammaplasty/ or breast implantation/ or scleroplasty/ or sex reassignment surgery/ or ulnar collateral ligament reconstruction/ or reoperation/ or second-look surgery/ or splenectomy/ or surgery, computer-assisted/ or robotic surgical procedures/ or symphysiotomy/ or thoracic surgical procedures/ or mediastinoscopy/ or pulmonary surgical procedures/ or bronchoscopy/ or lung transplantation/ or sternotomy/ or thoracoplasty/ or thoracoscopy/ or thoracic surgery, video-assisted/ or thoracostomy/ or thoracotomy/ or thymectomy/ |
| 5 | organ transplantation/ or kidney transplantation/ or liver transplantation/ or lung transplantation/ or pancreas transplantation/ or vascularized composite allotransplantation/ or facial transplantation/ or hand transplantation/ or replantation/ or corneal transplantation/ or epikeratophakia/ or keratoplasty, penetrating/ or skin transplantation/ or ultrasonic surgical procedures/ or lithotripsy/ or lithotripsy, laser/ or phacoemulsification/ or piezosurgery/ or urogenital surgical procedures/ or gynecologic surgical procedures/ or hysterectomy/ or trachelectomy/ or hysteroscopy/ or ovariectomy/ or salpingo-oophorectomy/ or salpingectomy/ or salpingostomy/ or sterilization, tubal/ or uterine artery embolization/ or uterine myomectomy/ or vulvectomy/ or sterilization, reproductive/ or sterilization reversal/ or vasovasostomy/ or vasectomy/ or urologic surgical procedures/ or cystectomy/ or cystoscopy/ or cystotomy/ or nephrectomy/ or nephroureterectomy/ or nephrolithotomy, percutaneous/ or nephrotomy/ or nephrostomy, percutaneous/ or ureteroscopy/ or urinary diversion/ or cystostomy/ or ureterostomy/ or urologic surgical procedures, male/ or orchiectomy/ or orchiopexy/ or penile implantation/ or prostatectomy/ or "transurethral resection of prostate"/ |
| 6 | specialties, surgical/ or colorectal surgery/ or general surgery/ or neurosurgery/ or orthognathic surgery/ or surgery, plastic/ or thoracic surgery/ |
| 7 | recovery room/ or operating rooms/ |
| 8 | su.fs. |
| 9 | (abdominal surgery or breast surgery or cancer surgery or ear nose throat surgery or elective surgery or emergency surgery or endocrine surgery or eye surgery or general surgery or geriatric surgery or "head and neck surgery" or major surgery or minimally invasive surgery or minor surgery or nanosurgery or neurosurgery or open surgery or orthopedic surgery or pediatric surgery or newborn surgery or pelvis surgery or plastic surgery or prophylactic surgical procedure or reconstructive surgery or reoperation or robot assisted surgery or second look surgery or segmentectomy or telesurgery or thorax surgery or transplantation or urologic surgery or transsphenoidal surgery or abdominal wall closure or biliary tract surgery or gastrointestinal surgery or hernioplasty or herniorrhaphy or herniotomy or laparoscopy or laparotomy or liver surgery or omentectomy or omentoplasty or peritoneum lavage or spleen surgery or bile duct reconstruction or biliary tract drainage or cholecystectomy or cholecystotomy or choledochotomy or endoscopic papillotomy or vater papillotomy or bile duct bypass or gallbladder drainage or percutaneous transhepatic drainage or cholecystostomy or choledochoduodenostomy or choledochojejunostomy or hepatojejunostomy or pancreaticojejunostomy or portoenterostomy or gastrointestinal surgery or antireflux operation or anus surgery or bariatric surgery or intestine surgery or pancreas surgery or stomach surgery or vagotomy or stomach fundoplication or Belsey fundoplication or Dor fundoplication or Nissen fundoplication or Thal fundoplication or Toupet fundoplication or transoral incisionless fundoplication or Rossetti fundoplication or anoplasty or hemorrhoidectomy or biliopancreatic bypass or gastric banding or sleeve gastrectomy or laparoscopic sleeve gastrectomy or appendectomy or colon surgery or colorectal surgery or endoscopic polypectomy or enterostomy or ileum pouch or intestine anastomosis or intestine bypass or intestine resection or intestine transplantation or polypectomy or rectum surgery or colon anastomosis or colon resection or colostomy or hemicolectomy or sigmoidectomy or total colon resection or colorectal anastomosis or hartmann procedure or proctocolectomy or cecostomy or colostomy or continent ileostomy or duodenostomy or ileostomy or jejunostomy or colon anastomosis or colorectal anastomosis or gastroduodenostomy or gastroenterostomy or gastrojejunostomy or ileoanal anastomosis or ileorectal anastomosis or jejunoileostomy or jejunum interposition or portoenterostomy or rectum anastomosis or Roux Y anastomosis or Roux-en-Y gastric bypass or ileum bypass or jejunoileal bypass or rectum resection or small intestine resection or rectum abdominoperineal resection or rectum anterior resection or total mesorectal excision or ileum resection or intestine graft or ileum graft or proctopexy or pull through operation or rectum anastomosis or pancreas duct ligation or pancreas transplantation or pancreatectomy or pancreaticoduodenectomy or pancreaticojejunostomy or kidney pancreas transplantation or pancreas islet transplantation or distal pancreatectomy or gastrectomy or gastric bypass surgery or gastropexy or gastroplasty or gastrostomy or gastrotomy or percutaneous endoscopic gastrostomy or pyloromyotomy or pyloroplasty or pylorus ligation or stomach pouch or gastrectomy Billroth I or gastrectomy Billroth II or partial gastrectomy or stomach antrum resection or total stomach resection or highly selective vagotomy or selective vagotomy or truncus vagotomy or hand assisted laparoscopy or laparoendoscopic single site surgery or laparoscopic cholecystectomy or laparoscopic surgery or hepatic artery ligation or hepatocyte transplantation or liver resection or liver transplantation or hemihepatectomy or liver lobectomy or partial hepatectomy or liver graft or spleen transplantation or splenectomy or (breast reconstruction or mastectomy or breast augmentation or breast reduction or lumpectomy or partial mastectomy or prophylactic mastectomy or radical mastectomy or simple mastectomy or subcutaneous mastectomy or extended radical mastectomy or modified radical mastectomy or cytoreductive surgery or lymph node dissection or pelvis exenteration or pelvis lymphadenectomy or ear surgery or endoscopic sinus surgery or ethmoidectomy or nose surgery or parotidectomy or throat surgery or ear reconstruction or labyrinthectomy or mastoidectomy or middle ear reconstruction or middle ear surgery or middle ear ventilation or myringoplasty or myringotomy or stapes surgery or transtympanic micropressure treatment or tympanoplasty or stapedectomy or stapedotomy or stapes mobilization or dacryocystorhinostomy or endoscopic endonasal surgery or nose reconstruction or nose septum reconstruction or adenoidectomy or larynx surgery or pharyngectomy or pharyngostomy or pharynx reconstruction or tonsillectomy or uvulopalatopharyngoplasty or laryngectomy or laryngoplasty or adenotonsillectomy or palatine tonsillectomy or adrenalectomy or castration or gonadectomy or orchiectomy or parathyroid transplantation or parathyroidectomy or thyroid surgery or subtotal thyroidectomy or thyroidectomy or thyroparathyroidectomy or capsulotomy or cataract extraction or cornea surgery or dacryocystorhinostomy or eye enucleation or eye evisceration or eyelid reconstruction or glaucoma surgery or iridectomy or iridotomy or lens implantation or lensectomy or orbital exenteration or refractive surgery or scleroplasty or sclerotomy or strabismus surgery or transpupillary thermotherapy or vitreoretinal surgery or capsulorhexis or extracapsular cataract extraction or intracapsular cataract extraction or phacoemulsification or zonulolysis or keratectomy or keratoplasty or keratotomy or laser epithelial keratomileusis or laser refractive surgery or limbal stem cell transplantation or photorefractive keratectomy or radial keratotomy or filtering operation or sclerectomy or sclerostomy or trabeculectomy or trabeculoplasty or trabeculotomy or viscocanalostomy or keratomileusis or small incision lenticule extraction or retina surgery or vitrectomy or radial optic neurotomy or retina detachment surgery or retina macula translocation or retinotomy or retinopexy or sclera buckling procedure or pars plana vitrectomy or craniofacial surgery or neck dissection or oral surgery or cervicoplasty or cranioplasty or face surgery or orbit reconstruction or palatoplasty or facial nerve surgery or forehead flap or lip reconstruction or maxillofacial surgery or orthognathic surgery or alveolar bone grafting or genioplasty or mandible osteotomy or mandibular advancement or maxilla osteotomy or sagittal split ramal osteotomy or Le Fort osteotomy or Le Fort I osteotomy or Le Fort II osteotomy or Le Fort III osteotomy or gingivectomy or gingivoplasty or glossectomy or mandible reconstruction or mandible resection or maxilla resection or parotidectomy or preprosthetic surgery or sinus floor augmentation or alveolar ridge augmentation or alveolectomy or alveoloplasty or vestibuloplasty) or (auditory brain stem implantation or nerve surgery or neuroendoscopy or neuronavigation or skull surgery or spinal cord surgery or sympathectomy or vagotomy or ventriculostomy or facial nerve surgery or nerve decompression or nerve reconstruction or neurectomy or neurolysis or neurotomy or nerve anastomosis or nerve elongation or nerve suture or nerve transplantation or nerve graft or vidian neurectomy or axotomy or radial optic neurotomy or brain surgery or cerebrovascular surgery or craniectomy or cranioplasty or craniotomy or decompression surgery or brain decompression or corpus callosum transection or decompressive craniectomy or hemispherectomy or hypophysectomy or pallidotomy or psychosurgery or stereotaxic surgery or temporal lobectomy or thalamotomy or transsphenoidal surgery or transsphenoidal hypophysectomy or cerebrovascular surgery or brain artery bypass or cerebral revascularization or extraintracranial anastomosis or brain decompression or brain ventricle peritoneum shunt or cerebrospinal fluid drainage or cerebrospinal fluid shunting or decompressive craniectomy or microvascular decompression or nerve decompression or spinal cord decompression or cordotomy or discectomy or foraminotomy or laminectomy or laminoplasty or rhizotomy or spinal cord decompression or percutaneous discectomy or dorsal rhizotomy or lumbar sympathectomy or highly selective vagotomy or selective vagotomy or truncus vagotomy or third ventriculostomy or ventriculocisternostomy or amputation or bone resection or bone transplantation or cartilage transplantation or cementoplasty or "closed reduction (procedure)" or distraction osteogenesis or fasciotomy or foot surgery or fracture treatment or hand surgery or hemipelvectomy or joint surgery or leg lengthening or ligament surgery or limb salvage or muscle resection or muscle transposition or "open reduction (procedure)" or osteotomy or periosteum transplantation or spine surgery or tendon surgery or amputation stump or disarticulation or finger amputation or foot amputation or hand amputation or limb amputation or thumb amputation or traumatic amputation or arm amputation or leg amputation or above knee amputation or below knee amputation or knee amputation or bone graft or bone allograft or bone patellar tendon bone graft or fibula graft or orbit implant or rib graft or sinus floor augmentation or synthetic bone graft or cartilage graft or meniscal transplantation or percutaneous vertebroplasty or kyphoplasty or closed fracture reduction or closed luxation reduction or bunionectomy or foot amputation or fracture fixation or fracture reduction or percutaneous vertebroplasty or traction therapy or closed fracture reduction or open fracture reduction or finger amputation or finger arthroplasty or hand amputation or hand reconstruction or thumb amputation or finger replacement or finger replantation or thumb reconstruction or arthrodesis or arthrolysis or arthroplasty or arthroscopic surgery or arthrotomy or bursectomy or capsular release or chondroplasty or hip surgery or joint capsulotomy or knee surgery or shoulder surgery or synovectomy or ankle arthrodesis or epiphysiodesis or sacroiliac joint fusion or shoulder arthrodesis or spine fusion or subtalar arthrodesis or anterior spine fusion or posterior lumbar interbody fusion or posterior spine fusion or spondylodesis or ankle arthroplasty or elbow arthroplasty or finger arthroplasty or hip arthroplasty or knee arthroplasty or replacement arthroplasty or revision arthroplasty or shoulder arthroplasty or ankle replacement or elbow replacement or finger replacement or acetabuloplasty or hip replacement or hip hemiarthroplasty or total hip replacement or knee replacement or total knee arthroplasty or one-stage revision or two-stage revision or reverse shoulder arthroplasty or shoulder replacement) or (abrasion arthroplasty or arthroscopic debridement or arthroscopic resection or arthroscopic resection or chondrocyte implantation or mosaicplasty or thermal chondroplasty or knee ligament surgery or anterior cruciate ligament reconstruction or posterior cruciate ligament reconstruction or meniscal surgery or meniscal repair or meniscal transplantation or meniscectomy or shoulder hemiarthroplasty or ulnar collateral ligament reconstruction or open luxation reduction or femur intertrochanteric osteotomy or femur osteotomy or fibula osteotomy or fibulotibial osteotomy or hip osteotomy or metatarsal osteotomy or pelvis osteotomy or tibia osteotomy or tibia proximal osteotomy or periosteum graft or intradiscal electrothermal therapy or spine stabilization or total disc replacement or tendon transfer or tendon transplantation or tenodesis or tenotomy or tendon graft or gynecologic surgery or pelvis exenteration or pelvis lymphadenectomy or anterior intravaginal slingplasty or clitoroplasty or colporrhaphy or anterior colporrhaphy or posterior colporrhaphy or colposuspension or colpotomy or defibulation or female sterilization or ovariectomy or salpingooophorectomy or female sterilization reversal or hymenoplasty or posterior intravaginal slingplasty or pubovaginal sling procedure or sacrocolpopexy or uterine tube surgery or uterus surgery or vagina reconstruction or vulvectomy or salpingoplasty or salpingostomy or uterine tube sterilization or salpingectomy or uterine tube coagulation or uterine tube ligation or cervicectomy or "dilatation and curettage" or endometrium ablation or hysterectomy or myomectomy or uterine cervix cerclage or uterine cervix conisation or abdominal hysterectomy or hysterotomy or radical hysterectomy or vaginal hysterectomy or esthetic surgery or body contouring or liposuction or lipoabdominoplasty or lipectomy or reimplantation or limb replantation or sex transformation or skin surgery or abdominoplasty or rhytidoplasty or skin transplantation or skin expansion or skin graft or composite skin graft or free skin graft or full thickness skin graft or skin allograft or skin autograft or skin flap or pedicled skin flap or skin flap survival or skin island flap or skin transposition flap or split thickness skin graft or tissue expansion or tissue flap or adipofascial flap or anterolateral thigh flap or chimeric flap or deep inferior epigastric perforator flap or deltopectoral flap or fasciocutaneous flap or forehead flap or free tissue graft or gracilis flap or inferior gluteal artery perforator flap or inguinal flap or island flap or lateral arm flap or latissimus dorsi flap or muscle flap or myocutaneous flap or neurovascular island flap or osteocutaneous flap or parascapular flap or paraumbilical perforator flap or perforator flap or radial forearm flap or scapular flap or transverse rectus abdominis musculocutaneous flap or vertical rectus abdominis musculocutaneous flap or Z plasty or surgical flaps or tendon reconstruction) or (anastomosis or cauterization or cerclage or chemosurgery or coagulation surgery or computer assisted surgery or robotic surgical procedure or cryosurgery or cryocoagulation or device removal or dissection or electrosurgery or electrocoagulation or endoscopic surgery or excision or ganglionectomy or pinealectomy or wide excision or exeresis or fenestration or implantation or prosthesis implantation or incision or laser surgery or laser coagulation or lithotomy or lobectomy or metastasis resection or microsurgery or morcellation or myotomy or ostomy or radical resection or radiosurgery or radiofrequency ablation or bronchial thermoplasty or sphincteroplasty or sphincterotomy or surgical drainage or ultrasound surgery or wedge resection or esophagus surgery or lung surgery or bronchoplasty or lung lobectomy or lung resection or lung transplantation or oleothorax or pleurodesis or pneumolysis or tracheobronchial toilet or pleurectomy or rib graft or rib resection or sternotomy or thoracocentesis or thoracoplasty or thoracostomy or thoracotomy or thorax drainage or thorax wall reconstruction or thymus surgery or thymectomy or thymus transplantation or thymus graft or trachea surgery or trachea reconstruction or trachea resection or tracheostomy or tracheotomy or video assisted thoracoscopic surgery or male genital system surgery or circumcision or male sterilization or castration or orchiectomy or vasectomy or male sterilization reversal or orchidopexy or penis amputation or prostate surgery or laser prostatectomy or prostatectomy or robot-assisted prostatectomy or radical retropubic prostatectomy or transurethral electrovaporization or "transurethral incision of the prostate" or transurethral microwave thermotherapy or transurethral needle ablation or transurethral resection or varicocelectomy or vasovasostomy or sterilization reversal or urinary tract surgery or bladder surgery or bladder reconstruction or cystectomy or cystostomy or cystotomy or ureteroneocystostomy or kidney surgery or kidney transplantation or kidney allograft or kidney autotransplantation or kidney graft or kidney pancreas transplantation or nephrectomy or bilateral nephrectomy or nephron sparing surgery or nephroureterectomy or partial nephrectomy or heminephrectomy or radical nephrectomy or uninephrectomy or nephrolithotomy or percutaneous nephrolithotomy or nephrolithotripsy or nephrostomy or percutaneous nephrostomy or nephrotomy or pyelolithotomy or pyeloplasty or pyelotomy or ureter surgery or nephroureterectomy or ureter reimplantation or ureter replacement or ureteral stenting or ureterolithotomy or ureteroneocystostomy or ureterosigmoidostomy or ureterostomy or ureteroureterostomy or urethra surgery or suburethral sling procedure or suprapubic arc sling procedure or tension-free vaginal tape procedure or transobturator tape procedure or urethroplasty or urethrotomy or urinary diversion or continent urinary reservoir or ileal conduit or ureteroneocystostomy or ureterosigmoidostomy)).mp. |
| 10 | (Clitoridectomy or Vaginectomy or Penectomy or Posthectomy or Hemicorporectomy or Hypophysectomy or Amygdalohippocampectomy or Corpectomy or Facetectomy or Gangliectomy or Neurectomy or Stapedectomy or Mastoidectomy or Trabeculectomy or Glossectomy or Esophagectomy or Rhinectomyor Pneumonectomy or Hypophysectomy or Thymectomy or Splenectomy or Lymphadenectomy or Adenectomy or Coccygectomy or Ostectomy or Astragalectomy or Frenectomy or Gingivectomy or Lobectomy or Myomectomy or Panniculectomy or Hepatoportoenterostomy or Urostomy or Hymenotomy or Episiotomy or Laminotomy or Foraminotomy or Axotomy or Vagotomy or Myringotomy or Tenotomy or Escharotomy or Arthrotomy or Pyloromyotomy or Sinusotomy or Cricothyrotomy or Bronchotomy or Thyrotomy).mp. |
| 11 | (operating room* or operating theat* or operation room* or operation theat* or PACU room* or PACU ward* or post-an?esthe* care or postan?esthe* care or recovery ward* or recovery room* or an?esthe* recovery or operative* or intraoperative* or perioperative* or postoperative* or surgical* or operation or peroperative* or surger* or surgeon* or neurosurg* or postsurg*).mp. |
| 12 | or/1-11 |
| 13 | mortality/ or "cause of death"/ or fatal outcome/ or hospital mortality/ or mortality, premature/ or survival rate/ |
| 14 | Survival/ or survival analysis/ or kaplan-meier estimate/ |
| 15 | vital statistics/ or life expectancy/ or life tables/ or morbidity/ |
| 16 | (death* or mortalit* or fatal* or survival* or morbidit* or kaplan meier).mp. |
| 17 | or/13-16 |
| 18 | 12 and 17 |
| 19 | surgical procedures, operative/mo or ablation techniques/mo or cautery/mo or cryosurgery/mo or corneal surgery, laser/mo or lithotripsy, laser/mo or anastomosis, surgical/mo or gastroenterostomy/mo or pancreaticojejunostomy/mo or salpingostomy/mo or vasovasostomy/mo or bariatric surgery/mo or lipectomy/mo or lipoabdominoplasty/mo or biopsy/mo or curettage/mo or "dilatation and curettage"/mo or vacuum curettage/mo or cytoreduction surgical procedures/mo or debridement/mo or decompression, surgical/mo or microvascular decompression surgery/mo or device removal/mo or digestive system surgical procedures/mo or anastomosis, roux-en-y/mo or appendectomy/mo or biliopancreatic diversion/mo or cholecystectomy/mo or cholecystectomy, laparoscopic/mo or cholecystostomy/mo or choledochostomy/mo or portoenterostomy, hepatic/mo or sphincterotomy, endoscopic/mo or sphincterotomy, transduodenal/mo or colectomy/mo or proctocolectomy, restorative/mo or endoscopy, digestive system/mo or cholangiopancreatography, endoscopic retrograde/mo or endoscopy, gastrointestinal/mo or balloon enteroscopy/mo or double-balloon enteroscopy/mo or single-balloon enteroscopy/mo or colonoscopy/mo or sigmoidoscopy/mo or duodenoscopy/mo or endoscopic mucosal resection/mo or esophagoscopy/mo or gastroscopy/mo or proctoscopy/mo or transanal endoscopic surgery/mo or transanal endoscopic microsurgery/mo or pyloromyotomy/mo or enterostomy/mo or cecostomy/mo or colostomy/mo or duodenostomy/mo or ileostomy/mo or jejunostomy/mo or esophagectomy/mo or esophagoplasty/mo or esophagostomy/mo or fundoplication/mo or gastrectomy/mo or gastropexy/mo or gastroplasty/mo or gastrostomy/mo or heller myotomy/mo or hemorrhoidectomy/mo or hepatectomy/mo or jejunoileal bypass/mo or lateral internal sphincterotomy/mo or liver transplantation/mo or pancreas transplantation/mo or pancreatectomy/mo or pancreaticoduodenectomy/mo or proctectomy/mo or elective surgical procedures/mo or electrosurgery/mo or endocrine surgical procedures/mo or adrenalectomy/mo or castration/mo or orchiectomy/mo or ovariectomy/mo or salpingo-oophorectomy/mo or hypophysectomy/mo or parathyroidectomy/mo or pinealectomy/mo or thyroidectomy/mo or fasciotomy/mo or (hemostasis, surgical/mo or keratectomy/mo or corneal surgery, laser/mo or keratectomy, subepithelial, laser-assisted/mo or keratomileusis, laser in situ/mo or photorefractive keratectomy/mo or laparotomy/mo or ligation/mo or lymph node excision/mo or neck dissection/mo or mastectomy/mo or metastasectomy/mo or microsurgery/mo or minimally invasive surgical procedures/mo or monitoring, intraoperative/mo or myotomy/mo or pyloromyotomy/mo or sphincterotomy/mo or obstetric surgical procedures/mo or colposcopy/mo or colpotomy/mo or culdoscopy/mo or cesarean section/mo or hysteroscopy/mo or hysterotomy/mo or neurosurgical procedures/mo or anterior temporal lobectomy/mo or brain tissue transplantation/mo or cerebrospinal fluid shunts/mo or ventriculoperitoneal shunt/mo or ventriculostomy/mo or craniotomy/mo or decompressive craniectomy/mo or trephining/mo or denervation/mo or parasympathectomy/mo or vagotomy/mo or sympathectomy/mo or ganglionectomy/mo or cordotomy/mo or nerve crush/mo or rhizotomy/mo or foraminotomy/mo or hypophysectomy/mo or laminectomy/mo or laminoplasty/mo or microvascular decompression surgery/mo or stereotaxic techniques/mo or radiosurgery/mo) or (ophthalmologic surgical procedures/mo or blepharoplasty/mo or dacryocystorhinostomy/mo or eye enucleation/mo or eye evisceration/mo or sclerostomy/mo or trabeculectomy/mo or iridectomy/mo or orbit evisceration/mo or refractive surgical procedures/mo or cataract extraction/mo or capsulorhexis/mo or phacoemulsification/mo or corneal transplantation/mo or descemet stripping endothelial keratoplasty/mo or epikeratophakia/mo or keratoplasty, penetrating/mo or corneal surgery, laser/mo or keratectomy, subepithelial, laser-assisted/mo or keratomileusis, laser in situ/mo or photorefractive keratectomy/mo or keratotomy, radial/mo or lens implantation, intraocular/mo or posterior capsulotomy/mo or scleroplasty/mo or scleral buckling/mo or vitrectomy/mo or vitreoretinal surgery/mo or oral surgical procedures/mo or apicoectomy/mo or gingivectomy/mo or gingivoplasty/mo or glossectomy/mo or jaw fixation techniques/mo or orthognathic surgical procedures/mo or osteotomy, le fort/mo or osteotomy, sagittal split ramus/mo or orthopedic procedures/mo or acetabuloplasty/mo or amputation/mo or disarticulation/mo or hemipelvectomy/mo or arthrodesis/mo or spinal fusion/mo or arthroplasty/mo or anterior cruciate ligament reconstruction/mo or bone-patellar tendon-bone grafting/mo or arthroplasty, replacement/mo or hemiarthroplasty/mo or total disc replacement/mo or posterior cruciate ligament reconstruction/mo or arthroscopy/mo or joint capsule release/mo or bone lengthening/mo or ilizarov technique/mo or osteogenesis, distraction/mo or bone transplantation/mo or cementoplasty/mo or vertebroplasty/mo or kyphoplasty/mo or diskectomy/mo or diskectomy, percutaneous/mo or fracture fixation/mo or closed fracture reduction/mo or fracture fixation, internal/mo or fracture fixation, intramedullary/mo or open fracture reduction/mo or laminectomy/mo or limb salvage/mo or meniscectomy/mo or osteotomy/mo or alveolar bone grafting/mo or synovectomy/mo or tendon transfer/mo or tenodesis/mo or tenotomy/mo or traction/mo or ulnar collateral ligament reconstruction/mo or ostomy/mo or cystostomy/mo or enterostomy/mo or cecostomy/mo or colostomy/mo or duodenostomy/mo or ileostomy/mo or jejunostomy/mo or esophagostomy/mo or gastrostomy/mo or middle ear ventilation/mo or nephrostomy, percutaneous/mo or pharyngostomy/mo or thoracostomy/mo or tracheostomy/mo or ureterostomy/mo) or (otorhinolaryngologic surgical procedures/mo or adenoidectomy/mo or laryngectomy/mo or laryngoplasty/mo or laryngoscopy/mo or nasal surgical procedures/mo or rhinoplasty/mo or neck dissection/mo or otologic surgical procedures/mo or auditory brain stem implantation/mo or cochlear implantation/mo or endolymphatic shunt/mo or fenestration, labyrinth/mo or mastoidectomy/mo or middle ear ventilation/mo or myringoplasty/mo or ossicular replacement/mo or stapes surgery/mo or transtympanic micropressure treatment/mo or tympanoplasty/mo or pharyngectomy/mo or pharyngostomy/mo or tonsillectomy/mo or tracheostomy/mo or tracheotomy/mo or pelvic exenteration/mo or perioperative care/mo or intraoperative care/mo or postoperative care/mo or perioperative period/mo or intraoperative period/mo or operative time/mo or postoperative period/mo or anesthesia recovery period/mo or pneumonectomy/mo or prophylactic surgical procedures/mo or prosthesis implantation/mo or maxillofacial prosthesis implantation/mo or mandibular prosthesis implantation/mo or reconstructive surgical procedures/mo or abdominoplasty/mo or lipoabdominoplasty/mo or acetabuloplasty/mo or dermatologic surgical procedures/mo or blepharoplasty/mo or rhytidoplasty/mo or skin transplantation/mo or herniorrhaphy/mo or limb salvage/mo or lipectomy/mo or mammaplasty/mo or breast implantation/mo or scleroplasty/mo or sex reassignment surgery/mo or ulnar collateral ligament reconstruction/mo or reoperation/mo or second-look surgery/mo or splenectomy/mo or surgery, computer-assisted/mo or robotic surgical procedures/mo or symphysiotomy/mo or thoracic surgical procedures/mo or mediastinoscopy/mo or pulmonary surgical procedures/mo or bronchoscopy/mo or lung transplantation/mo or sternotomy/mo or thoracoplasty/mo or thoracoscopy/mo or thoracic surgery, video-assisted/mo or thoracostomy/mo or thoracotomy/mo or thymectomy/mo) or (organ transplantation/mo or kidney transplantation/mo or liver transplantation/mo or lung transplantation/mo or pancreas transplantation/mo or vascularized composite allotransplantation/mo or facial transplantation/mo or hand transplantation/mo or replantation/mo or corneal transplantation/mo or epikeratophakia/mo or keratoplasty, penetrating/mo or skin transplantation/mo or ultrasonic surgical procedures/mo or lithotripsy/mo or lithotripsy, laser/mo or phacoemulsification/mo or piezosurgery/mo or urogenital surgical procedures/mo or gynecologic surgical procedures/mo or hysterectomy/mo or trachelectomy/mo or hysteroscopy/mo or ovariectomy/mo or salpingo-oophorectomy/mo or salpingectomy/mo or salpingostomy/mo or sterilization, tubal/mo or uterine artery embolization/mo or uterine myomectomy/mo or vulvectomy/mo or sterilization, reproductive/mo or sterilization reversal/mo or vasovasostomy/mo or vasectomy/mo or urologic surgical procedures/mo or cystectomy/mo or cystoscopy/mo or cystotomy/mo or nephrectomy/mo or nephroureterectomy/mo or nephrolithotomy, percutaneous/mo or nephrotomy/mo or nephrostomy, percutaneous/mo or ureteroscopy/mo or urinary diversion/mo or cystostomy/mo or ureterostomy/mo or urologic surgical procedures, male/mo or orchiectomy/mo or orchiopexy/mo or penile implantation/mo or prostatectomy/mo or "transurethral resection of prostate"/mo) or (specialties, surgical/mo or colorectal surgery/mo or general surgery/mo or neurosurgery/mo or orthognathic surgery/mo or surgery, plastic/mo or thoracic surgery/mo) or (recovery room/mo or operating rooms/mo) |
| 20 | 18 or 19 |
| 21 | ((POSSUM or Surgical Outcome Risk or NZRISK or APACHE or ACS NSQIP or ASA PS or ASA or SRS or Charlson Comorbidity or SAPS or BHOM or Surgical Apgar or IRIS or SMPM or mE-PASS or MSQC or CORES or ESAS or SURPAS or POTTER or VASQIP or Surgical Risk or Child-Turcotte-Pugh or CTP or MELD) and (tool* or score* or scale* or model* or index or class* or calculat*)).mp. |
| 22 | 20 or 21 |
| 23 | Health Services, Indigenous/ |
| 24 | oceanic ancestry group/ |
| 25 | Indigenous Peoples/ |
| 26 | (Ayabakan or Aiabakan or Bakanu or Baganu or Bakanh or Ayapathu or Aiabadu or Aiyabotpoo or Jabuda or Koka Ai ebadu or Aiebadu or Koko Aiebadu or Kikahiabilo or Bakanh or Alawa or Alaua or Allawa or Allaua or Allua or Allowa or Alowa or Leealowa or Kalawa or Kallaua or Allowiri or Allaura or Galleewo or Alura or Allura or Hallura or Nallura or Jaminjung or lillup or Alyawarre or Iliaura or Illiaura or Iljaura or Ilyaura or Ilyowra or Illyowra or Illura or Aliawara or Alyawara or Alyawarra or Ilawara or Jaljuwara or Yalyuwara or Alyawarr or Amangu or Emangu or Amandyo or Amarak or Amarag or Amuruk or Amurag or Amurrak or Ngamurak or Ngamurag or Umuriu or Umoreo or Amijangal or Amijangal or Anaywan or Nowan or Enni won or Yenniwon or Ee na won or En nee win or Eneewin or Inuwan or Inuwon or Neeinuwon or Enuin or Nganyayawana or Andakerebina or Antakiripina or Undejerebina or Andeberegina or Walwallie or Andakerebina or Andegerebenha or Adnyamathanha or Kuyani or Wailpi or Yadliaura or Pilatapa or Pangkala or Ankamuti or Goomkoding or Yukamakundji or Amkomti or Ondaima or Oiyamkwi or Apukwi or Anggamudi or Anmatyerre or Nmatjera or Unmatjera or Inmatjera or Anmatjara or Urmitchee or Janmadjara or Janmatjiri or Yanmedjara or Yandmadjari or Anmatjera or Antakirinja or Antakerinya or Antakerrinya or Andagirinja or Andagarinja or Andekerinja or Andegilliga or Andigirinji Antingari or Andigari Anjirigna or Andgari Antigari or Antegarina or Unterregerrie or Ngonde or Tangara or Yandairunga or Njuntundjara or Andagerinja or Antekerrepinhe or Andergerebenha or Andigerinya or Antekarinya or Antikirinya or Araba or Aripa or Ngariba or Arabana or Ngarabana or Arabuna or Arrabunna or Arrabonna or Arubbinna or Arapina or Arapani or Urapuna or Urabuna or Urabunna or Urroban or Wangarabana or Wongkurapuna or Wangarabunna or Jendakarangu or Yendakarangu or Arakwal or Arakwal or Naiang or Cool al or Kahwul or Njung or Nyung or Kogung or Yawkum yore or Jawjumeri or Arrernte or Aranda or Aranta or Arrente or Arunda or Arunta or Arranda or Arinta or Arrinda or Urrundie or Herinda or Arrundta or Wonggaranda or Urrundie or Ilpma or Ulpma or Paroola or Wongkatjeri or Arnga or Woljamidi or Woljamiri or Molyamidi or Kuluwara or Kuluwaran or Guluwarin or Kolaia or Arawari or Arawodi or Atjinuri or Adjinadi or Itinadjana or Itinadyana or Itinadyand or Nedgulada or Imatjana or Awabakal or Awaba or Awabagal or Kuringgai or Ninyowa or Kuri or Awarai or Warai or Warei or Warrai or Awarrai or Awarra or Awinmul or Awinnmull or Awinmil or Awngthim or Badjalang or Badjalang or Buggul or Bandjalang or Widje or Woomargou or Bundjalung or Badjiri or Badjidi or Badjeri or Baddyeri or Byierri or Baderi or Poidgerry or Badjedi or Budjari or Poidgerryygbadhje or Baiali or Byellee or Bieli or Byellel or Orambul or Urambal or Bayali or Baiyungu or Baijungo or Baijungu or Baiong or Baiung or Biong or Paiunggu or Bayungu or Palyungu or Payungu or Bailgu or Bailko or Bailgu or Pailgu or Pailgo or Baljgu or Balju or Palgu or Bailju or Bailgo or Balgu or Boolgoo or Pulgoe or Mangguldulkara or Paljarri or Palyku or Palku or Bakanambia or Wanbara or Wambara or Lamalama or Mukinna or Balardong or Balardung or Banbai or Banbai or Bahnbi or Athnbi or Gumbaynggirr or Bandjigali or Bandjin or Barada or Baradha or Baranbinja or Baranbinja or Barren binya or Parran binye or Burranbinya or Burrunbinya or Barrumbinya or Burranbinga or Burrabinya or Barranbinya or Barapa Barapa or Baraparapa or Burrabura-ba or Baraba-baraba or Barraba-barraba or Bareber or Burrappa or Burrapper or Bureba or Burabura or Boora boora or Burapper or Baraba Baraba or Barbaram or Mbabaram or Badimaya or Barimaia or Bardi or Barda or Baada or Baardi or Barindji or Barrengee or Beriait or Berri ait or Paur or Paroo or Bpaaroo or Bpaaroon je or Barkindji or Barkinji or Barkinjee or Barkunjee or Bahkunji or Pakindji or Bakindji or Bahkunjy or Parkungi or Parkengee or Parkingee or Bakandi or Bargunji or Kurnu or Wimbaja or Barkungee or Barna or Barunggam or Barungguan or Batjala or Badtjala or Beriguruk or Perrigurruk or Erei or Beriguruk or Limilngan or Bidawal or Bidia or Birria or Bidjigal or Bediagal or Bigambul or Bilingara or Bilinara or Bilinurra or Bilyanarra or Bilyanurra or Plinara or Pillenurra or Billianera or Bulinara or Bringara or Boonarra or Binbinga or Binbingha or Binbinka or Pinbinga or Leepitbinga or Bing Binga or Bindal or Bindjali or Bingongina or Bin gongina or Bugongidja or Bingongina or Binigura or Pinikura or Biria or Birpai or Birpai or Birripai or Birrpiri or Brippai or Bripi or Birrapee or Biripi or Bitjara or Brabralung or Brabiralung or Brabuwooloong or Braiakaulung or Brayakuloong or Bratauolung or Bratowooloong or Bugulmara or Bukurnidja or Buluwai).mp. |
| 27 | (Bunganditj or Buandig or Bundjalung or Boonwurrung or Bunurong or Boonwurrung or Burarra or Anbara or Marawuraba or Madia or Maringa or Gunadba or Gunaidbe or Gidjingali or Barera or Barara or Baurera or Burera or Barea or Anbarra or Bwgcolman or Daii or Taii or Dalwango or Dalwongu or Darlwongo or Dhalwangu or Djawark or Djarlwag or Dainggati or Djangadi or Dang getti or Danghetti or Danggadi or Dhangatty or Thangatty or Thangatti or Dangati or Yuungai or Burugardi or Boorkutti or Nulla Nulla or Amberu or Himberrong or Daingatti or Dhan Gadi or Dainggatti or Dalabon or Buan or Buwan or Boun or Ngalkbon or Ngalkbun or Dalla or Dalulinta or Dangbon or Gundangbon or Dangbun or Dangbar or Gumauwurk or Danggali or Dangu or Yirgala or Yolngu or Darambal or Darumbal or Darkinjang or Darkinjang or Darginjang or Darkinung or Darknung or Darug or Daruk or Dharruk or Dharrook or Darrook or Dharug or Dharung or Dharawal or Diakui or Hiyakuy or Djikai or Jikai or Tchikai or Dijogoi or Dieri or Djabugandji or Djabugay or Djabugandji or Tjapukai or Djagaraga or Djakunda or Djalakuru or Jalakuru or Iwaidja or Djamindjung or Tjamindjung or Kaminjung or Jaminjang or Djamunjun or Jaminjung or Djamundon or Djamadjong or Murinyuwen or Murinyuwan or Djamindjung or Djangu or Djanga or Djankun or Djargurd Wurrung or Djaru or Jaru or Dja Dja Wurrung or Djerait or Tjerait or Cherait or Cherite or Sherait or Jeerite or Scherits or Tjiras or Tjerratj or Djerimanga or Djeramanga or Jermangel or Kanambre or Kolobre Waak or Wulna or Woolna or Woolnah or Woolner or Wulnar or Wolna or Woolua or Djilamatang or Djinang or Jandjinang or Jandjinung or Jakaula or Yandisha or Yandjinung or Yandjining or Yandjinang or Djinnang or Djinang or Djinhang or Milingimbi or Wulllakki or Wulaki or Ullaki or Wulagi or Balmbi or Balmawi or Barlmawi or Manjarngi or Manyarrngi or Munarngo Manarrngu or Djinba or Djimba or Jinba or Outjanbah or Gunalbingu or Ganalbwingu or Kurkamarnapia or Djirbalngan or Djiru or Djirubal or Djiwali or Jirwali or Djowei or Kumertuo or Djowei or Limilngan or Djugun or Jukun or Doolboong or Duduroa or Duulngari or Dunghutti or Dhangadi or Boorkutti or Burgadi or Burugardi or Dainggati or Dainiguid or Dang-getti or Dangadi or Dangati or Danggadi or Danggetti or Danghetti or Dhangatty or Djaingadi or Nulla Nulla or Tang gette or Tangetti or Thangatti or Duwal or Duwal Dhuwal or Murngin or Wulamba or Miwuyt or Balamumu or Barlamomo or Malag or Marlark or Arrawiya or Banjarrpuma or Bilmandji Dhurili or Durilji or Duwala or Duala Duwala or Murngin or Wulamba or Eora or Eo ra or Ea ora or Iora or Yo ra or Kameraigal or Camera gal or Cammera or Gweagal or Bedia-mangora or Gouia-gul or Wanuwangul or Cadigal or Gadigal or Kadigal or Erawirung or Ewamin or Agwamin or Gaari or Gari Gaari or Iwaidja or Gadjalivia or Gadjalivia Gajalivia or Gudjalibi or Gudalavia or Gudjaliba or Gadjalibi or Gadjalibir or Burara or Gambalang or Gunbalang or Gambalang or Gunbulan or Walang or Gunbalang or Gandangara or Gandangara or Gundungurra or Gundungari or Gundanora or Gurragunga or Burragorang or Gundungurra or Garrwa or Karawa or Garawa or Geawegal or Keawaikal or Geawagal or Geawa gal or Garewagal or Gwea gal or Giya or Giabal or Gkuthaarn or Khutant or Kareldi or Kutanda or Goeng or Goenpul or Minjerribah or Goreng goreng or Gureng Gureng or Curang Curang or Curang gurang or Goeng Goonine or Goorang Goorang or Goori or Goorie or Gurang or Gurang Gurang or Kooranga or Koreng Koreng or Koren or Korenggoreng or Gudjal or Gugu Badhun or Gulidjan or Gulngai or Gunai or Ganai or Gunnai or Kurnai Koori or Koorie or Kurnai or Gumbaynggirr or Gumbaynggirr or Wadjar Gunavidji or Gunavidji Gunaviji or Gunawitji or Gunabidji or Gunabwidji or Gunjibidji or Witji or Gunibidji or Gunditjmara or Gournditch mara or Dhauwurd wurrung or Dhawurdwurrung or Djab Wurrung or Djabwurrung or Gundidjmara or Kilcarer Gundidj or Worn Gundidj or Gungorogone or Gungorogone Gungoragone or Gungorologni or Gungarawoni or Gungurulgungi or Gungurugoni or Gubi Gubi or Kabi Kabi or Gunwinggu or Gunwingo or Wengi or Wengej or Gundeidjeme or Gundeidjepmi or Gunwingu or Kulunglutji or Kulunglutchi or Gundeijeme or Margulitban or Unigangk or Urnigangg or Koorungo or Neinggu or Mangaridji Mangeri or Gurindji or Guugu Yimidhirr or Idindji or Ilba or Yilba or Ildawongga or Inawongga or Yinhawangka or Indindji or Yidinji or Indjibandi or Yindjibarndi or Indjilandji or Indjilandji Indjilindji or Injilinji or Intjilatja or Inggarda or Yinggarda or Ingura or Wanindilaugwa or Andiljaugwa or Andilyaugwa or Wani-Ndiljaugwa or En Indiljaugwa or Amakurupa or Andilagwa or Lamadalpu or Awarikpa or Warnindilyakwa or Iningai or Irukandji or Yirrganydji or Irakanji or Yirkandji or Yirkanji or Yirgay or Yettkie or Illagona or Wongulli or Dungara or Tingaree or Dungarah or Dingal or Ithu or Iwaidja or Jiwadja or Jiwadja or Juwudja or Iwajia or Eiwaja or Eaewardja or Eaewarga or Uwaidja or Unalla or Limbakaraja or Limba Karadjee or Iwaiji or Tarula or Jardwadjali or Jaadwa Jadawadjali or Jaako or Jaara or Djadjawurung or Jabirr Jabirr or Jabirrjabirr or Djaberadjabera or DjaberrDjaberr or Dyaberdyaber).mp. |
| 28 | (Jabba Jabba or Jaburara or Jaburrara or Yaburara or Burrara or Jadira or Jadliaura or Jagalingu or Yagalingu or Jagara or Jaitmathang or Jaitmatang or Jalanga or Jambina or Yambina or Jandruwanta or Yandruwandha or Jangaa or Yanga or Jangga or Janggal or Gananggalanda or Jangkundjara or Yankuntjatjara or Jangman or Yangman or Janjula or Jardwadjali or Jarijari or Jarildekald or Jaroinga or Bularnu or Jarowair or Jathaikana or Jaudjibaia or Yauraworka or Yawrawarka or Jawi or Djawi or Djaui or Jawoyn or Tjauen or Djouan or Djauun or Jawin or Chau an or Tweinbol or Adowen or Djawin or Djawun or Djauwung or Charmong or Djauan or Jawuru or Yawuru or Jeidji or Yiiji or Jeithi or Jeithi or Yeidthee or Pikkolatpan or Wiradjuri or Jeljendi or Yarluyandi or Jeteneru or Jetimarala or Jiegara or Jiegera or Yiegara or Jeigir or Yegera or Youngai or Jungai or Jilngali or Jiman or Yiman or Jinigudira or Jinwum or Minwum or Jirandali or Yirandali or Jirjoront or Yir Yoront or Jitajita or Jitajita or Ita ita or Ithi ithi or Eethie eethie or Eethee Ethee or Yetho or Yit tha or Yitsa or Tjuop or Yitha Yitha or Jokula or Ganggalida or Juat or Yuat or Juipera or Yuwi or Jukambal or Jukambal or Jukambil or Yukambal or Yukambul or Yukambil or Yacambal or Yookumbul or Yookumbil or Yoocumbill or Ukumbil or Yookumble or Yoocomble or Ucumble or Yukumba or Jukambe or Jukul or Julaolinja or Jumu or Junggor or Jungkurara or Jupagalk or Jupangati or Yupangathi or Juru or Yuru or Kaantju or Kaanju or Kabalbara or Gabalbara or Kabikabi or Gubbi Gubbi or Kadjerong or Kaiabara or Kaiadilt or Gayardilt or Kairi or Gayiri or Kaititja or Kakadu or Gagudju or Kalaako or Kalali or Kullilla or Kalamaia or Kalaamaya or Kalibal or Kalibal or Murwillumbah or Moorung moobar or Kalibamu or Kalkadunga or Kalkadoon or Kalkatungu or Kalkutungu or Galgadungu or Kalkutung or Galgaduun or Kambure or Gamberre or Kambuwal or Kamilaroi or Kamilarai or Kamilari or Kamilroi or Kamilrai or Kamularoi or Kaameelarrai or Komleroy or Gamilaroi or Kahmilaharoy or Kamilary or Gumilori or Gummilroi or Ghummilarai or Cumilri or Kimilari or Kamil or Comleroy or Camel Duahi or Yauan or Tjake or Gamilaraay or Goomeroi or Yuwaalaraay or Gamilaraay or Yorta Yorta or Kandju or Kaneang or Kaniyang or Kangulu or Gangulu or Kanolu or Karadjari or Karajarri or Karaman or Karangpurru or Karanguru or Karangura or Karanja or Kareldi or Gkuthaarn or Kuthant or Kareldi or Kutanda or Garandi or Karundi or Karrandee or Karendala or Karenggapa or Karengappa or Karrengappa or Kurengappa or Kariara or Kariyarra or Karingbal or Garingbal or Kartudjara or Mardu or Karuwali or Katubanut or Gadubanud or Kaurareg or Kaurna or Coorna or Gaurna or Koornawarra or Nantuwara or Nantuwaru or Nganawara or Meljurna or Meyukattanna or Kawadji or Kawambarai or Kareingi or Kerinma or Karinma or Garengema or Garnghes or Kinenekinene or Kianigane or Keramin or Kemendok or Pintwa or Jungeegatchere or Kayimai or Kaytetye or Kartetye or Kartiji or Keytej or Katish or Keiadjara or Keinjan or Keramai or Kirrae or Girai wurrung or Kitabal or Kidabal or Dijabal or Kitta bool or Kitabool or Kitapul or Gidabul or Gidjoobal or Kuttibul or Noowidal or Kitja or Kija or Guwa or Koamu or Kooma or Koara or Kuwarra or Koenpal or Koinjmal or Guwinmal or Kokangol or Kokatha or Kokata or Kokatha Mula or Kokatja or Googatha or Koknar or Kokobididji or Kokobujundji or Kokoimudji or Kokojawa or Kokojelandji or Kuku yalanji or Kokokulunggur or Kokomini or Kokonjekodi or Kokopatun or Kokopera or Koko bera or Kokowalandja or Kokowara or Kokowarra or Kolakngat or Konbudj or Gonbudj or Konejandi or Gooniyandi or Kongabula or Gungabula or Kongkandji or Koreng Goreng or Korenggoreng or Gureng Gureng or Korindji or Gurindji or Kotandji or Ngandji or Krauatungalung or Kujani or Kuyani or Kukatj or Kukatj or Marago or Gudadj or Gudadji or Gugady or Gugatj or Kokatj or Kukatji or Kukatyi or Konggada or Kukatja or Kokatja or Gugadja or Kukati or Kuku Yulanji or Kuku Yulangi or Gugu Yulanji or Kuuku yani or Noolulgo or Kurnu or Gunu or Guerno or Kornu or Kornoo or Kuno or Guno or Gunu or Kulumali or Kumbainggiri or Kumbainggiri or Kumbainggeri or Kumbaingir or Kumbaingeri or Kumbangerai or Koombanggary or Koombainga or Coombangree or Coombyngura or Gumbaingar or Gunbaingar or Guinbainggri or Belingen or Nimboy or Woolgoolga or Orara or Gumbainggir or Gumbaynggir or Kunapa or Kundjey mi or Kungadutji or Kungarakan or Kungarakany or Kunggara or Kurtjar or Kunggari or Gunggari or Gungarri or Kungkalenja or Kunindiri or Gunindiri or Kunja or Kurrama or Kurama or Gurama or Kerama or Karama or Korama or Kormama or Jana ri or Jawunmara or Kureinji or Kareingi or Kerinma or Karinma or Garengema or Garnghes or Kinenekinene or Kianigane or Keramin or Kemendok or Pintwa or Jungeegatchere or Kuringgai or Awabakal or Kurnai or Ganai or Gunai or Gunnai or Kurung or Kutjal or Kutjala or Kuuku ya'u or Kuungkari or Kuwema or Kwantari or Kwarandji or Kwatkwat or Kwiambal or Kweembul or Quieumble or Queenbulla or Ngarabal or Kwini or Lairmairrener or Lama Lama or Lanima or Larrakia or Larakia or Larrakeyah or Larakya or Lardiil or Lardil or Latjilatji or Latie Latie or Lotiga or Luritja or Loritja or Kukatja or Gugadja or Luthigh).mp. |
| 29 | (Madjandji or Madngela or Madoitja or Maduwongga or Magatige or Maiawali or Maijabi or Mayi Yapi or Maikudunu or Mayi Kutuna or Maikulan or Mayi Kulan or Maithakari or Mayi Thakurti or Malak Malak or Malantji or Malgana or Malkana or Malgaru or Maljangapa or Maljangpa or Malya napa or Mulya napa or Mulya nappa or Mullia arpa or Malynapa or Maljapa or Malyapa or Maljangaba or Karikari or Bulali or Bulali or Malyangaba or Malngin or Malpa or Mamu or Morruburra or Manbarra or Mandandanji or Mandara or Manthi or Mandjildjara or Mandjindja or Mangarla or Mangala or Mangarai or Mangarayi or Mungaria or Marra or Maranganji or Margany or Maranunggu or Maraura or Mareawura or Mareaura or Marowra or Marowera or Marraa Warree or Marrawarra or Waimbio or Wimbaja or Wiimbaio or Berlko or Ilaila or Barkindji or Marditjali or Mardudunera or Martuthunira or Mariamo or Maridan or Maridjabin or Marijedi or Marimanindji or Marramaninjsji or Maringar or Marringarr or Marinunggo or Marithiel or Marrithiyel or Mariu or Marrago or Martu or Mardu or Marulta or Matuntara or Maung or Maia or Mbewum or Mbeiwum or Mbukarla or Meintangk or Menthajangal or Meru or Ngaiawang or Ngawait or Nganguruku or Erawirung or Mian or Miyan or Milpulo or Milpulko or Mailpurlgu or Mamba or Danggali or Mimungkum or Minang or Mingin or Minjambuta or Minjungbal or Minjangbal or Minyung or Minyowa or Gendo or Gando Minjang or Gandowal or Ngandowul or Cudgingberry or Miriwung or Miriwoong or Miriuwong or Mirning or Mitaka or Mithaka or Mitjamba or Mbara or Miwa or Morowari or Murawari or Murawarri or Murrawarri or Muruworri or Muruwurri or Murueri or Moorawarree or Marawari or Marawara or Muruwari or Mpalitjanh or Muluridji or Muragan or Murinbata or Murrinh patha or Muringura or Murngin or Murunitja or Muthimuthi or Muti Muti or Mutte Mutte or Matimati or Madi madi or Mataua or Moorta or Matthee matthee or Bakiin or Madi Madi or Madhi Madhi or Maruara or Yita Yida or Tati Dadi or Mutjati or Mutpura or Mudburra or Mutumui or Nakako or Nakara or Nganawongka or Nhanta or Nangah or Nangatadjara or Nyanganyatyara or Nangatara or Nanggikorongo or Nanggumiri or Ngan giwumirri or Narangga or Narinari or Nari Nari or Naualko or Nawalko or Ngunnhalgu or Unelgo or Bungyarlee or Wampandi or Wampangee or Wombungee or Barundji or Nauo or Nawu or Nawagi or Nyawaygi or Ngadadjara or Ngatatjara or Ngaanyatjarra or Ngadjunmaia or Ngatjumay or Ngadjuri or Nadjuri or Ngaiawang or Ngaiawongga or Ngaku or Niungacko or Dainggatti or Dunghutti or Ngalakan or Ngalea or Ngalia or Ngaliwuru or Ngaluma or Ngarluma or Ngamba or Ngambar or Ngeunbah or Biripi or Ngameni or Ngamini or Ngandangara or Ngandi or Ngan gikurunggurr or Nganguruku or Ngarabal or Ngarabul or Ngarrabul or Narbul or Marbul or Ngaralta or Ngardi or Ngarti or Ngardok or Ngarigo or Ngarego or Ngarago or Garego or Currak da bidgee or Ngarigu or Ngarrugu or Ngarroogoo or Murring or Bemeringal or Guramal or Gurmal or Bradjerak or Bombala or Menero or Cooma or Ngarinjin or Ngarinyin or Ngarinman or Ngarkat or Ngargad or Ngarla or Ngarlawongga or Ngalawangka or Ngarrindjeri or Raminyeri or Ramindjeri or Narrinyeri or Ngaro or Ngathokudi or Ngatjan or Ngaun or Ngawun or Ngawait or Ngewin or Nggamadi or Ngintait or Ngiyambaa or Ngemba or Ngoborindi or Nguburinji or Ngolibardu or Ngolokwangga or Ngombal or Ngumbarl or Ngormbur or Ngombur or Ngugi or Ngulungbara or Ngunawal or Ngunuwal or Ngoonawawal or Wonnawal or Nungawal or Molonglo or Gurungada or Ngundjan or Kunjen or Ngurawola or Ngurelban or Ngurraiillam or Nguri or Ngurlu or Ngurunta or Niabali or Nimanburu or Ninanu or Njakinjaki or Nyaki Nyaki or Njamal or Nyamal or Njangamarda or Nyangumarda or Njikena or Nyikina or Njulnjul or Nyul Nyul or Njunga or Njuwathai or Noala or Nhuwala or Nhuwala palyku or Nokaan or Noongar or Njunga or Nyoongar or Nyungar or Norweilemil or Nuenonne or Nukunu or Nunggubuju or Nunggubuyu or Nunukul or Oitbi or Worla or Olkolo or Ombila or Ongkarango or Unggarangi or Ongkomi or Unggumi or Otati or Pakadji or Pandjima or Banjima or Pangerang or Pangkala or Banggarla or Paredarerme or Parundji or Paruindji or Paruindi or Paruinji or Paroinge or Barundji or Barungi or Bahroonjee or Baroongee or Bahroongee or Barrengee or Parooinge or Barunga or Paiyungu or Peerapper or Peramangk or Perrakee or Pibelmen or Bibbulman or Pilatapa or Pirlatapa or Pindiini or Pindjarup or Pinjareb or Pinjarup or Pini or Pintupi or Pintubi or Bindubi or Bindibu or Bindubu or Pitapita or Pitta Pitta or Pitjantjatjara or Pitjantjara or Pitjandjara or Pitjara or Bidjara or Pongaponga or Pontunj or Portaulun or Potaruwutj or Potidjara or Punaba or Punuba or Puneitja or Punthamara or Pyemmairre or Rakkaia or Ramindjeri or Rembarunga or Rembarnga or Ringaringa or Rungarungawa or Pila Nguru or Tagalag or Takalak or Tagoman or Taior or Thaayorre or Talandiji or Thalanyji or Talandji or Tanganekald or Targari or Tharrgari or Taribelang or Tatitati or Dadi Dadi or Tatungalung or Tatungoloong or Taungurong).mp. |
| 30 | (Tedei or Tenma or Tepiti or Teppathiggi or Tepiti or Tharawal or Darawal or Carawal or Turawal or Thurawal or Thurrawal or Thurrawall or Turuwal or Turuwul or Turrubul or Tutuwull or Ta ga ry or Thaua or Thawa or Tauaira or Thurga or Thoorga or Durga or Dhurga or Tharawal or Tadera manji or Guyanagal or Guyangal yuin or Murring or Katungal or Baianga or Paienbera or Thereila or Thiin or Tirari or Dhirari or Tjalkadjara or Tjalkanti or Tjapukai or Tjapwurong or Djabwurung or Tjeraridjal or Tjial or Tjingili or Jingili or Tjongkandji or Tjungundji or Tjupany or Tjuroro or Jurruru or Tommeginne or Toogee or Totj or Tulua or Tunuvivi or Tiwi or Tyerremotepanner or Umbindhamu or Barungguan or Umede or Umida or Umpila or Ombila or Undanbi or Unjadi or Uutaalnganu or Wadere or Wadikali or Wadigali or Wadja or Wadjigu or Wadjabangai or Wadjalang or Dharawala or Wadjari or Watjarri or Wagiman or Wagoman or Wailpi or Wakabunga or Wakaja or Wakaya or Wakaman or Wakara or Wakawaka or Waka Waka or Walangama or Walbanga or Thurga or Thoorga or Bugellimanji or Bargalia or Moruya or Walgalu or Walgadu or Wolgal or Wolgah or Tumut or Guramal or Gurmal or Waljen or Walmadjari or Walmatjarri or Walmbaria or Walpiri or Warlpiri or Walu or Waluwara or Warluwarra or Wambaia or Wambaya or Wanamara or Wunumara or Wandandian or Wandandian or Tharumba or Kurialyuin or Murraygaro or Wandarang or Wandjira or Wangaaybuwan or Wongaibon or Ngemba or Ngeumba or Ngiumba or Wangan or Wongi or Wangatha or Wanji or Waanyi or Wanjiwalku or Weyneubulkoo or Wonipalku or Wanyabalku or Wonjimalku or Pernowie or Pernowrie or Kongait or Tongaranka or Wandjiwalgu or Wanjuru or Wanman or Warakamai or Waramanga or Warumungu or Wardal or Wardaman or Wardandi or Wargamaygan or Warakamai or Wariangga or Warriyangga or Warkawarka or Warki or Warlmanpa or Warray or Warungu or Warwa or Wathaurung or Wathawurrung or Wada Warrung or Wathaurong or Watiwati or Wadi Wadi or Watta or Waveroo or Wawula or Weilwan or Wailwan or Wayilwan or Weiwan or Wilwan or Wallwan or Wailwun or Waal won or Wile wile or Wali or Waljwan or Ngiumba or Weilwan or Wailwan or Wembawemba or Wambawamba or Wamba Wamba or Womba or Weumba or Waamba or Waimbiwaimbi or Gourrmjanyuk or Gorrmjanyuk or Wemba Wemba or Wenamba or Wenambal or Wembria or Weraerai or Wiraiarai or Weraiari or Wirri wirri or Wirraarai or Warlarai or Wolroi or Wolleri or Waholari or Wolaroo or Walarai or Juwalarai or Walari or Wolaroi or Woolaroi or Ginniebal or Whadjuk or Wadjuk or Whajook or Wadjug or Wajuk or Widi or Widjabal or Noowidal or Nowgyujul or Waibra or Ettrick or Watji or Watchee or Wiilman or Weelman or Wikapatja or Wikatinda or Wikepa or Wik kalkan or Wiknatanja or Wikianji or Mimungkum or Wikmean or Wikampama or Wikapatja or Wikatinda or Wikepa or Wikianji or Wik kalkan or Wikmean or Wikmunkan or Wiknantjara or Wiknatanja or Wilawila or Wilingura or Wilyakali or Wiljakali or Wiljali or Wiljagali or Bo arli or Bulali or Wilyali or Winduwinda or Winda Winda or Wiradjuri or Wiradyuri or Wiradhuri or Wiraduri or Wiradjeri or Wirrajerre or Wiradhari or Wirra dhari or Wirradhurri or Wirraijuri or Wirrathuri or Wiradthuri or Wiradtheri or Wirathere or Wira durei or Wira shurri or Wirradgerry or Woradjeri or Wooradjeri or Woorajuri or Woradjerg or Wirotheree or Wiratheri or Wi ra jer ree or Wirrai Durhai or Wirangu or Wirdinja or Wiri or Wirngir or Wodiwodi or Woddi Woddi or Tharawal or Wogait or Worgait or Wooptang or Wongaibon or Wongai bun or Wongabon or Wonghibone or Wonjhibon or Wonjibone or Wongi bone or Wonghi or Wungai or Wuzai or Wozai or Mudall or Wangaaybuwan or Wongkadjera or Wongkamala or Wangkamana or Wongkanguru or Wangkangurru or Wongkumara or Wangkumara or Wonnarua or Wonnuaruah or Wannerawa or Wonarura or Wonnah or Worimi or Warrimee or Warramie or Gadang or Kattang or Kutthung or Guttahn or Cottong or Wattung or Watthungk or Kutthack or Gingai or Gringai or Gooreenggai or Worora or Wotjobaluk or Wotjoballuk or Wergaia or Wudjari or Wulgurukaba or Wulili or Wuli wuli or Wulpura or Wulwulam or Wunambal or Wunambul or Wuningargk or Wurango or Wurundjeri or Wirundjeri or Woiworung or Woiwurrong or Wuthathi or Yamatji or Amangu or Badimia or Yamatji Marlpa or Baiyungu or Budina or Gnulli or Malgana or Naaguja or Thudgari or Yugunga Nya or Wajarri Yamatji or Yanda or Janda or Yuguurtuu or Yadhaykenu or Yanyuwa or Anula or Janjula or Yanula or Yanuwa or Yarra Yarra or Yaygirr or Yaegl or Yiman or Yiwara or Yolngu or Dangu or Yorta Yorta or Bangerang or Jotijota or Yotayota or Yoorta or Gunbowers or Gunbowerooranditchgoole or Ngarrimouro or Arramouro or Woollathura or Yota Yota or Kailtheban or Wollithiga or Ulupna or Kwat Kwat or Yalaba Yalaba or Nguaria iiliam wurrung or Kamilaroi or Yuggera or Jagara or Yuin or Yuwin or Djiringanj or Dyirringan or Jeringin or Juwin or Thaua or Yulparitja or Nangatara or Yuwaalaraay or Yualarai or Yualloroi or Yowaleri or Uollaroi or Youallerie or Yualari or Yualai or Yerraleroi or Yowalri or Euahlayi or Juwaljai or Yuwalyai or Wallarai or Wolleroi or Walleri or Wollaroi or Noongahburrahs or Yuwaaliyaay or Ualarai or Murri or Murrdi or Gorrie or Wangai or Nunga or Anangu or Yapa or Bininj or Anindilyakwa or Palawa or Palawah or Pallawah or Parna).mp. |
| 31 | (Amata or Angurugu or Arnhem Land or Badu Island or Bamaga or Binjari or Papulankutja or Borroloola or Brewarrina or Cherbourg or Coen or Djarindjin Lombadina or Doomadgee or Erub Island or Galiwin'ku or Gapuwiyak or Goondiwindi or Gununa or Ngurupai or Iama Island or Imangara or Injinoo or Jigalong or Jilkminggan or Kalkaringi or Kalkarindji or Daguragu or Kalumburu or Koonibba or Kowanyama or Kunawarritji or Maningrida or Mapoon or Mapuru or Maralinga Tjarutja or Mer Island or Mimili or Murrin Bridge or Mutitjulu or Napranum or Nepabunna or Nipapanha or Ngukurr or Palm Island or Bwgcolman or Papunya or Warrumpi or Parnngurr or Pormpuraaw or Pukatja or Ernabella or Punmu or Ramingining or Raukkan or Saibai Island or Ltyentye Apurte or Seisia or Waiben island or Titjikala or Wadeye or Warmun or Warrwa or Weipa or Wilcannia or Wiluna or Woorabinda or Wugularr or Wurrumiyanga or Yalata or Yarrabah or Yirrkala or Yuendumu or Yungngora).mp. |
| 32 | (Torres Strait or Meriam or Guda Maluilgal or Maluilgal or Kulkalgal or Kaiwalagal or Saibailgal or Maluilgal or Kaurareg or Meriam Le or Mabuygiwgal or Boigu or Dauan or Saibai or Badu or Mabuiag or Dauan or Moa island or Mua island or Arkai or Yama or Iama or Warraber or Masig or Ugar island or Waiben or Waibene or Ngurupai or Narupai or Kirirri or Muralag or Gealug Island or Poruma Island or Mer Island).mp. |
| 33 | 26 or 27 or 28 or 29 or 30 or 31 or 32 |
| 34 | 33 and (aborigin* or native? or indigen* or tribe? or tribal or nation? or people? or ethnic*).mp. |
| 35 | 33 and (australia* or queensland* or victoria* or new south wales or tasmania* or northern territory or cape york peninsula or melbourne or sydney or darwin or alice springs or brisbane or adelaide or perth or hobart or canberra or arnhem).mp,cp,in. |
| 36 | 23 or 24 or 25 |
| 37 | 36 and (australia* or queensland* or victoria* or new south wales or tasmania* or northern territory or cape york peninsula or melbourne or sydney or darwin or alice springs or brisbane or adelaide or perth or hobart or canberra or arnhem).mp,cp,in. |
| 38 | (first people* or first nation*).mp. and (australia* or queensland* or victoria* or new south wales or tasmania* or northern territory or cape york peninsula or melbourne or sydney or darwin or alice springs or brisbane or adelaide or perth or hobart or canberra or arnhem).mp,cp,in. |
| 39 | (native adj (communit* or settlement* or village? or reservation? or reserve? or clan? or band? or confederac* or nation? or people? or population?)).mp. and (australia* or queensland* or victoria* or new south wales or tasmania* or northern territory or cape york peninsula or melbourne or sydney or darwin or alice springs or brisbane or adelaide or perth or hobart or canberra or arnhem).mp,cp,in. |
| 40 | (native adj (m#n or wom#n or boy? or girl? or male? or female? or adolescent? or youth? or person? or adult? or child* or people* or nation? or tribe? or tribal or band or bands)).mp. and (australia* or queensland* or victoria* or new south wales or tasmania* or northern territory or cape york peninsula or melbourne or sydney or darwin or alice springs or brisbane or adelaide or perth or hobart or canberra or arnhem).mp,cp,in. |
| 41 | (aborigin* or indigen*).mp. and (australia* or queensland* or victoria* or new south wales or tasmania* or northern territory or cape york peninsula or melbourne or sydney or darwin or alice springs or brisbane or adelaide or perth or hobart or canberra or arnhem).mp,cp,in. |
| 42 | ((native? or tribe? or tribal) adj1 australia*).mp. |
| 43 | torres strait.mp. |
| 44 | 34 or 35 or 37 or 38 or 39 or 40 or 41 or 42 or 43 |
| 45 | 22 and 44 |
| 46 | 21 and 44 |
| 47 | 45 or 46 |
| 48 | limit 47 to ed=20210517-20210630 |
| 49 | (operating room* or operating theat* or operation room* or operation theat* or PACU or an?esth* or perian?esth* or postan?esthe* or recovery ward* or recovery room* or operative* or intraoperati* or preoperati* or perioperati* or postoperati* or peroperative* or surgeon* or postsurg* or (surgery or surgeries or surgical or reconstructive or reoperation* or telesurger* or transplant* or laparoscop* or laparotom* or reconstruction or bypass or operation or endoscop* or resect* or excision* or dissect*)).mp. |
| 50 | cardiovascular surgical procedures/ or cardiac surgical procedures/ or arterial switch operation/ or cardiac valve annuloplasty/ or mitral valve annuloplasty/ or cardiomyoplasty/ or heart arrest, induced/ or circulatory arrest, deep hypothermia induced/ or heart bypass, right/ or fontan procedure/ or heart massage/ or heart transplantation/ or heart-lung transplantation/ or heart valve prosthesis implantation/ or transcatheter aortic valve replacement/ or maze procedure/ or myocardial revascularization/ or angioplasty, balloon, coronary/ or atherectomy, coronary/ or coronary artery bypass/ or coronary artery bypass, off-pump/ or internal mammary-coronary artery anastomosis/ or transmyocardial laser revascularization/ or norwood procedures/ or pericardial window techniques/ or pericardiectomy/ or pericardiocentesis/ or reperfusion/ or myocardial reperfusion/ or vascular surgical procedures/ or axillofemoral bypass grafting/ or embolectomy/ or balloon embolectomy/ or endarterectomy/ or endarterectomy, carotid/ or endovascular procedures/ or angioplasty/ or angioplasty, balloon/ or angioplasty, balloon, laser-assisted/ or angioplasty, laser/ or atherectomy/ or angioscopy/ or catheterization, central venous/ or catheterization, peripheral/ or catheterization, swan-ganz/ or percutaneous coronary intervention/ or limb salvage/ or peritoneovenous shunt/ or thrombectomy/ or mechanical thrombolysis/ or vascular grafting/ or arteriovenous shunt, surgical/ or blalock-taussig procedure/ or blood vessel prosthesis implantation/ or cerebral revascularization/ or portasystemic shunt, surgical/ or portacaval shunt, surgical/ or portasystemic shunt, transjugular intrahepatic/ or splenorenal shunt, surgical/ or venous cutdown/ |
| 51 | (abdominal procedure* or breast procedure* or elective procedure* or endocrine procedure* or eye procedure* or neck procedure* or major procedure* or minimally invasive procedure* or minor procedure* or nanosurgery or neurosurg* or orthop?edic procedure* or pelvi* procedure* or second look procedure* or segmentectom* or telesurg* or thora* procedure* or urologic* procedure* or transsphenoidal procedure* or abdominal wall closure* or biliary tract procedure* or gastrointestinal procedure* or hernioplast* or herniorrhaph* or herniotom* or liver procedure* or omentectom* or omentoplast* or peritoneum lavage or spleen procedure* or biliary tract drainage or cholecystectom* or cholecystotom* or choledochotom* or papillotom* or gallbladder drainage or percutaneous transhepatic drainage or cholecystostom* or choledochoduodenostom* or choledochojejunostom* or hepatojejunostom* or pancreaticojejunostom* or portoenterostom* or antireflux procedure* or anus procedure* or bariatric procedure* or intestin* procedure* or pancrea* procedure* or stomach procedure* or vagotom* or fundoplication* or anoplast* or hemorrhoidectom* or gastric banding or gastrectom* or appendectom* or colon procedure* or colorectal procedure* or polypectom* or enterostom* or ileum pouch* or anastomos* or rectum procedure* or colostom* or hemicolectom* or sigmoidectom* or hartmann procedure* or proctocolectom* or cecostom* or colostom* or duodenostom* or ileostom* or jejunostom* or gastroduodenostom* or gastroenterostom* or gastrojejunostom* or jejunoileostom* or jejunum interposition or portoenterostom* or intestin* graft* or ileum graft* or proctopexy or pull through procedure* or ligation* or pancreatectom* or pancreaticoduodenectom* or pancreaticojejunostom* or gastropexy or gastroplast* or gastrostom* or gastrotom* or pyloromyotom* or pyloroplast* or stomach pouch or laparoendoscopic* or hemihepatectom* or lobectom* or hepatectom* or liver graft* or splenectom* or mastectom* or breast augmentation or breast reduction or lumpectom* or cytoreductive procedure* or pelvi* exenteration or pelvi* lymphadenectom* or ear procedure* or sinus procedure* or ethmoidectom* or nose procedure* or parotidectom* or throat procedure* or labyrinthectom* or mastoidectom* or middle ear ventilation or myringoplast* or myringotom* or stapes procedure* or transtympanic micropressure treatment or tympanoplast* or stapedectom* or stapedotom* or stapes mobile#ation or dacryocystorhinostom* or endonasal procedure* or adenoidectom* or larynx procedure* or pharyngectom* or pharyngostom* or tonsillectom* or uvulopalatopharyngoplast* or laryngectom* or laryngoplast* or adenotonsillectom* or adrenalectom* or castration or gonadectom* or orchiectom* or parathyroidectom* or thyroid procedure* or thyroidectom* or thyroparathyroidectom* or capsulotom* or cataract extraction* or cataract procedure* or cornea* procedure* or dacryocystorhinostom* or eye enucleation* or eye evisceration* or glaucoma procedure* or iridectom* or iridotom* or lens implant* or lensectom* or orbital exenteration* or refractive procedure* or scleroplast* or sclerotom* or strabismus procedure* or transpupillary thermotherap* or vitreoretinal procedure* or capsulorhexis or phacoemulsification or zonulolysis or keratectom* or keratoplast* or keratotom* or keratomileusis or sclerectom* or sclerostom* or trabeculectom* or trabeculoplast* or trabeculotom* or viscocanalostom* or lenticule extraction or retina* procedure* or vitrectom* or retina* detachment procedure* or retina macula translocation* or retinotom* or retinopexy or sclera buckling procedure* or craniofacial procedure* or oral procedure* or cervicoplast* or cranioplast* or orbit procedure* or palatoplast* or facial nerve procedure* or forehead flap* or lip procedure* or maxillofacial surgery or orthognathic procedure* or alveolar bone graft* or genioplast* or osteotom* or mandibular advancement or gingivectom* or gingivoplast* or glossectom* or mandible procedure* or parotidectom* or preprosthetic procedure* or sinus floor augmentation* or alveolar ridge augmentation* or alveolectom* or alveoloplast* or vestibuloplast* or brain stem implant* or neuroendoscop* or neuronavigation or skull procedure* or spinal cord procedure* or sympathectom* or vagotom* or ventriculostom* or facial nerve procedure* or nerve decompression or neurectom* or neurolysis or neurotom* or nerve elongation or nerve suture or nerve graft* or axotom* or brain procedure* or cerebrovascular operation* or craniectom* or cranioplast* or craniotom* or decompression procedure* or brain decompression or corpus callosum transection or decompressive craniectom* or hemispherectom* or hypophysectom* or pallidotom* or psychosurgery or stereotaxic procedure* or thalamotom* or transsphenoidal procedure* or cerebrovascular procedure* or cerebral revasculari#ation or extraintracranial procedure* or brain ventricle peritoneum shunt or cerebrospinal fluid drainage or cerebrospinal fluid shunting or microvascular decompression or spinal cord decompression or cordotom* or discectom* or foraminotom* or laminectom* or laminoplast* or rhizotom* or lumbar sympathectom* or ventriculocisternostom* or amputat* or cementoplast* or closed reduction procedure* or distraction osteogenesis or fasciotom* or fracture treatment or hemipelvectom* or leg lengthening or ligament procedure* or limb salvage or muscle transposition or open reduction procedure* or spin* procedure* or tendon procedure* or disarticulation or bone graft* or bone allograft* or fibula graft* or orbit implant* or rib graft* or sinus floor augmentation or cartilage graft* or vertebroplast* or kyphoplast* or closed luxation reduction or bunionectom* or fracture fixation or fracture reduction or traction therapy or finger replacement or finger replantation or arthrodes#s or arthrolys#s or arthroplast* or arthroscopic procedure* or arthrotom* or bursectom* or capsular release or chondroplast* or hip procedure* or joint capsulotom* or knee procedure* or shoulder procedure* or synovectom* or epiphysiodesis or joint fusion or spin* fusion or lumbar interbody fusion or spondylodesis or ankle replacement or elbow replacement or finger replacement or acetabuloplast* or hip replacement or knee replacement or one-stage revision or two-stage revision or shoulder replacement or arthroscopic debridement or chondrocyte implant* or mosaicplast* or thermal chondroplast* or meniscal procedure* or meniscal repair or meniscectom* or hemiarthroplast* or open luxation reduction or periosteum graft* or intradiscal electrothermal therapy or spin* stabili#ation or total disc replacement or tendon transfer* or tenodesis or tenotom* or tendon graft* or gyn?ecologic* procedure* or pelvis exenteration or pelvis lymphadenectom* or anterior intravaginal slingplast* or clitoroplast* or colporrhaphy or anterior colporrhaphy or posterior colporrhaphy or colposuspension or colpotom* or defibulation or female sterili#ation or ovariectom* or salpingooophorectom* or hymenoplast* or posterior intravaginal slingplast* or pubovaginal sling procedure* or sacrocolpopexy or uterine tube procedure* or uter* procedure* or vagina* procedure* or vulvectom* or salpingoplast* or salpingostom* or uterine tube sterili#ation or salpingectom* or uterine tube coagulation or uterine tube ligation or cervicectom* or "dilatation and curettage" or endometrium ablation or hysterectom* or myomectom* or uterine cervix cerclage or uterine cervix conisation or hysterotom* or esthetic procedure* or body contouring or liposuction or lipoabdominoplast* or lipectom* or reimplantation or limb replantation or sex transformation or skin procedure* or abdominoplast* or rhytidoplast* or skin expansion or skin graft* or composite skin graft* or free skin graft* or full thickness skin graft* or skin allograft* or skin autograft* or skin flap* or skin island flap* or skin transposition flap* or split thickness skin graft* or tissue expansion or tissue flap* or adipofascial flap* or anterolateral thigh flap* or chimeric flap* or deep inferior epigastric perforator flap* or deltopectoral flap* or fasciocutaneous flap* or forehead flap* or free tissue graft* or gracilis flap* or inferior gluteal artery perforator flap* or inguinal flap* or island flap* or lateral arm flap* or latissimus dorsi flap* or muscle flap* or myocutaneous flap* or neurovascular island flap* or osteocutaneous flap* or parascapular flap* or perforator flap* or radial forearm flap* or scapular flap* or musculocutaneous flap* or Z plast* or cauteri#ation or cerclage or chemosurger* or coagulation procedure* or cryosurg* or cryocoagulation or device removal or electrosurg* or electrocoagulation or excision or ganglionectom* or pinealectom* or wide excision or exeresis or fenestration or implantation or prosthe* implantat* or incision or laser procedure* or laser coagulation or lithotom* or microsurgery or morcellation or myotom* or ostom* or radiosurger* or radiofrequency ablation* or bronchial thermoplast* or sphincteroplast* or sphincterotom* or surgical drainage or esophag* procedure* or oesophag* procedure* or lung procedure* or bronchoplast* or oleothorax or pleurodesis or pneumolysis or tracheobronchial toilet or pleurectom* or rib graft* or sternotom* or thoracocentesis or thoracoplast* or thoracostom* or thoracotom* or thorax drainage or thora* wall procedure* or thymus procedure* or thymectom* or thymus graft* or trachea* procedure* or trachea procedure* or tracheostom* or tracheotom* or thoracoscopic procedure* or genital system procedure* or circumcision or castration or orchiectom* or vasectom* or male sterili#ation or orchidopexy or prostat* procedure* or prostatectom* or transurethral electrovapori#ation or transurethral microwave thermotherap* or transurethral needle ablation or varicocelectom* or vasovasostom* or sterili#ation reversal or urinary tract procedure* or bladder procedure* or cystectom* or cystostom* or cystotom* or ureteroneocystostom* or kidney procedure* or kidney allograft* or kidney autotransplantation or kidney graft* or nephrectom* or nephron sparing procedure* or nephroureterectom* or heminephrectom* or uninephrectom* or nephrolithotom* or nephrolithotripsy or nephrostom* or nephrotom* or pyelolithotom* or pyeloplast* or pyelotom* or ureter procedure* or nephroureterectom* or ureter reimplant* or ureter replacement or ureteral stenting or ureterolithotom* or ureteroneocystostom* or ureterosigmoidostom* or ureterostom* or ureteroureterostom* or urethra* procedure* or suburethral sling procedure* or suprapubic arc sling procedure* or vaginal tape procedure* or transobturator tape procedure* or urethroplast* or urethrotom* or urinary diversion* or continent urinary reservoir or ileal conduit or ureteroneocystostom* or ureterosigmoidostom*).mp. |
| 52 | 49 or 50 or 51 |
| 53 | 17 and 44 and 52 |
| 54 | cardiovascular surgical procedures/mo or cardiac surgical procedures/mo or arterial switch operation/mo or cardiac valve annuloplasty/mo or mitral valve annuloplasty/mo or cardiomyoplasty/mo or heart arrest, induced/mo or circulatory arrest, deep hypothermia induced/mo or heart bypass, right/mo or fontan procedure/mo or heart massage/mo or heart transplantation/mo or heart-lung transplantation/mo or heart valve prosthesis implantation/mo or transcatheter aortic valve replacement/mo or maze procedure/mo or myocardial revascularization/mo or angioplasty, balloon, coronary/mo or atherectomy, coronary/mo or coronary artery bypass/mo or coronary artery bypass, off-pump/mo or internal mammary-coronary artery anastomosis/mo or transmyocardial laser revascularization/mo or norwood procedures/mo or pericardial window techniques/mo or pericardiectomy/mo or pericardiocentesis/mo or reperfusion/mo or myocardial reperfusion/mo or vascular surgical procedures/mo or axillofemoral bypass grafting/mo or embolectomy/mo or balloon embolectomy/mo or endarterectomy/mo or endarterectomy, carotid/mo or endovascular procedures/mo or angioplasty/mo or angioplasty, balloon/mo or angioplasty, balloon, laser-assisted/mo or angioplasty, laser/mo or atherectomy/mo or angioscopy/mo or catheterization, central venous/mo or catheterization, peripheral/mo or catheterization, swan-ganz/mo or percutaneous coronary intervention/mo or limb salvage/mo or peritoneovenous shunt/mo or thrombectomy/mo or mechanical thrombolysis/mo or vascular grafting/mo or arteriovenous shunt, surgical/mo or blalock-taussig procedure/mo or blood vessel prosthesis implantation/mo or cerebral revascularization/mo or portasystemic shunt, surgical/mo or portacaval shunt, surgical/mo or portasystemic shunt, transjugular intrahepatic/mo or splenorenal shunt, surgical/mo or venous cutdown/mo |
| 55 | 44 and 54 |
| 56 | 21 and 44 and 52 |
| 57 | 17 and 44 and 52 |
| 58 | 55 or 56 or 57 |
| 59 | 58 not 47 |
| 60 | 48 or 59 |
